# On the contributing role of the transmembrane domain for subunit-specific sensitivity of integrin activation

**DOI:** 10.1038/s41598-018-23778-5

**Published:** 2018-04-10

**Authors:** Giulia Pagani, Holger Gohlke

**Affiliations:** 1 Institute of Pharmaceutical and Medicinal Chemistry, Heinrich-Heine-Universität Düsseldorf, 40225 Düsseldorf, Germany; 2 John von Neumann Institute for Computing (NIC), Jülich Supercomputing Centre (JSC) & Institute for Complex Systems - Structural Biochemistry (ICS 6), Forschungszentrum Jülich GmbH, 52425 Jülich, Germany

## Abstract

Integrins are α/β heterodimeric transmembrane adhesion receptors. Evidence exists that their transmembrane domain (TMD) separates upon activation. Subunit-specific differences in activation sensitivity of integrins were reported. However, whether sequence variations in the TMD lead to differential TMD association has remained elusive. Here, we show by molecular dynamics simulations and association free energy calculations on TMDs of integrin α_IIb_β_3_, α_v_β_3_, and α_5_β_1_ that α_IIb_β_3_ TMD is most stably associated; this difference is related to interaction differences across the TMDs. The order of TMD association stability is paralleled by the basal activity of these integrins, which suggests that TMD differences can have a decisive effect on integrin conformational free energies. We also identified a specific order of clasp disintegration upon TMD dissociation, which suggests that the closed state of integrins may comprise several microstates. Our results provide unprecedented insights into a possibly contributing role of TMD towards subunit-specific sensitivity of integrin activation.

## Introduction

Integrins are a major class of heterodimeric adhesion receptors consisting of α and β subunits^1^ and are involved in the regulation of many biological events^2^. Each subunit is formed by a large extracellular domain (ectodomain) connected to a short cytoplasmic tail through a single transmembrane domain (TMD)^3^. In providing a physical link between the exterior and the interior of the cell, the TMD serves as a translator of mechanical and biochemical signals in both directions across the plasma membrane, leading to inside-out and outside-in signaling^4–6^. TMDs are directly involved in the mechanism of integrin activation^7^ in that the two transmembrane (TM) segments associate in the resting state^8–10^ and dissociate upon activation^11–14^. Structural features of the integrin TMD were revealed by nuclear magnetic resonance (NMR) structures^15–17^, biochemical data^18,19^, and electron microscopy^20–22^. Two structural elements, the inner and outer membrane clasps (IMC and OMC)^23^, were recognized as principal mediators of TMD assembly^24^, together with electrostatic interactions in the membrane-proximal region. However, despite these unifying principles, there is also evidence for specific ways of helix-helix association among different isoforms^25^, and it has remained unclear if and how these differences are linked to the subunit-specific sensitivity of integrin activation^26,27^: Integrin α_IIb_β_3_ has been described as basally inactive, in contrast to α_v_β_3_, which was found to be in the active state by default in certain cell types^28,29^, and β_1_ integrins, which are considered to be basally active^26^, with integrin α_5_β_1_ being among the most conformationally flexible integrins containing β_1_^27^. In addition, contradicting findings of NMR^24,30^ and *in vitro*^31^ *versus in vivo*^32^ studies with respect to a salt bridge interaction between α_IIb_-R995 and β_3_-D723 might also be related to specific TMD associations (see below for details).

The TMD of a subunit is formed by a short α-helix^33^, and two such TMDs are arranged in a right-handed coiled-coil conformation^16^ in the resting state of the integrin^9,10,34^. The structure of the TMD of α_IIb_β_3_ integrin^15^ revealed that the TM and membrane-proximal regions of the β_3_ subunit form a continuous helical structure that is tilted by ~25° with respect to the membrane normal^16,17^. In contrast, the α_IIb_ subunit is oriented in parallel to the membrane normal, breaks at G991, and bents towards the β_3_ TMD. This allows the dimer to be stabilized by the OMC and IMC^23^. The former is a G*XXX*G-like motif ^35^, while the latter is a highly conserved GFFKR motif ^36^, with the two Phe residues found in all α subunits^36^. Mutational studies^13^, disulphide scanning^11^ and Leu scanning^11^ experiments confirmed the importance of the OMC, whose alteration prevents correct helix packing and abolishes helix association^36^. However, different compositions of the G*XXX*G motif are found in α subunits, which makes it reasonable to hypothesize that the different OMC interfaces contribute to differential integrin activation. Likewise, the importance of the IMC, and, in particular, the two conserved Phe residues, in maintaining correct TMD packing and restraining integrin in the resting state has been shown^31,37^. In contrast, it has remained controversial whether α_IIb_-F992 engages β_3_-K716 in hydrogen bond formation to support IMC formation^17^, or whether K716 “snorkels” towards the lipid head groups^38^. Moreover, NMR spectroscopy^24^ revealed the presence of a salt bridge between α_IIb_-R995 and β_3_-D723 at the membrane-proximal region, in immediate vicinity of the IMC, whose functional role in restraining integrin in the inactive state has been demonstrated^24,30,39–41^. However, NMR structures in which both α_IIb_-F992 and α_IIb_-R995 point away from the β_3_ subunit, thus, not making any interactions with the β_3_ subunit, have also been determined^30^. Likewise, mutational studies in which the breaking of the R995-D723 salt bridge did not cause immediate integrin activation were reported^14,39^.

In order to provide insights at the atomistic level as to a potential influence of the TMD on the subunit-specific sensitivity of integrin activation, including the role of interactions across the IMC and OMC, here, we performed equilibrium molecular dynamics (MD) simulations in an explicit membrane environment of in total 9 μs length on the associated TMDs of integrins α_IIb_β_3_, α_v_β_3_, and α_5_β_1_, respectively, and potential of mean force (PMF) computations of TMD association of in total 3.5 μs sampling time, from which we derived association free energies. Our results show that the TMD of integrin α_IIb_β_3_ is most stably associated compared to α_v_β_3_ and α_5_β_1_. We relate these differences to particular interactions across the TMDs, with a focus on the different OMC compositions and an “OMC before IMC” order of clasp disintegration found for TMD dissociation. Our results provide unprecedentedly detailed and comparative insights into a possibly contributing role of the TMD towards subunit-specific sensitivity of integrin activation.

## Results

### Structural dynamics of the α_IIb_β_3_, α_v_β_3_, and α_5_β_1_ TMDs

**Figure 1:**
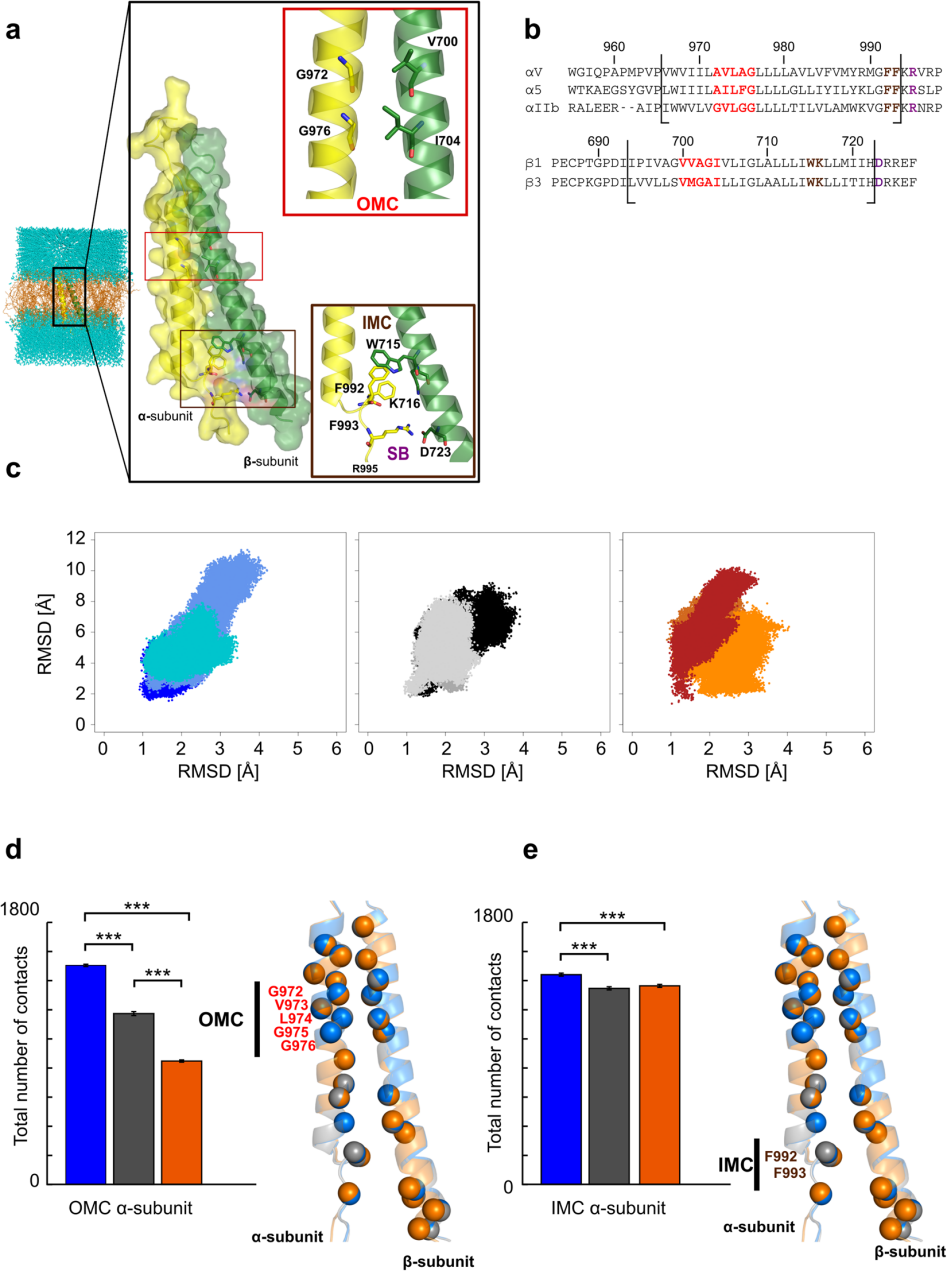
Structural integrity of the membrane-embedded helices throughout the MD simulations and contacts across the interface. (**a**) Exemplary simulation box generated to perform MD simulations including the NMR structure of α_IIb_β_3_ TMD (PDB ID 2K9J; cartoon and surface representation) with the position of the OMC (red box) and IMC (brown box) indicated; phospholipids are shown as orange sticks, and water layers as blue spheres. Close-up views of the box contents show essential residues mediating the clasps: α_IIb_-G972/G976 and β_3_-V700/I704 for the OMC, α_IIb_-F992/F993/R995 and β_3_-W715/K716/D723 for the IMC plus the putative salt bridge. (**b**) Sequence alignment of the α_IIb_β_3_, α_v_β_3_, α_5_β_1_ TMD sequences used to generate the homology models. G*XXX*G and GFFKR motifs are highlighted in red and brown, respectively, and R995/D723 residues in purple. Black bars indicate the TMD borders as reported in ref.^45^. (**c**) Two dimensional histograms of the RMSD values of all C_α_ atoms (ordinate values) and only those that are embedded in the membrane (abscissa values) (range of residues considered: P996-V1015 and D718-I747) calculated over three MD simulations. Blue, cornflowerblue, turquoiseblue colored dots represent α_IIb_β_3_ (MD simulations 1, 2 and 3); darkgrey, black, lightgrey α_v_β_3_; chocolate, orange, firebrick α_5_β_1_. (**d**,**e**) Histograms of the total number of overall contacts per residue at the OMC and IMC averaged over three MD simulations, with error bars showing the standard error of the mean (SEM; eq. 6) and stars indicating the statistical significance (see Methods section for definition). Here, only residues of the α subunit are shown (numbering refers to the α_IIb_ subunit). On the right of the plots, a superimposition of the α_IIb_β_3_, α_v_β_3_, and α_5_β_1_ TMDs in blue, grey, and orange, respectively, is shown. The C_α_ atoms of residues considered in the contact analysis are indicated as spheres, and the residues of the α subunit considered in the analyses are labeled.

In order to investigate at an atomistic level possible subunit-specific differences in the association of TMDs of integrins α_IIb_β_3_, α_v_β_3_, and α_5_β_1_, the three TMDs were subjected to all-atom MD simulations in an explicit membrane and solvent environment (Fig. 1a). Prior to that, we generated homology models of α_v_β_3_ and α_5_β_1_ TMDs using the NMR structure of α_IIb_β_3_ TMD (PDB ID 2K9J)^15^ as a template. To estimate the quality of the models, we used the QMEANBrane version^42^ of the QMEAN scoring function implemented in the QMEAN server^43^. QMEANBrane employs specifically trained potentials for three different segments (membrane, interface and soluble) in a transmembrane protein model to determine local (*i*.*e*., per residue) absolute quality estimates on the basis of a single model. With 1.0 as the optimal score, we found local scores of ~0.8 for the residues embedded in the membrane and ~0.6–0.8 when considering the overall structures (Fig. S1). The NMR structure showed very similar QMEANBrane scores, suggesting a sufficient local structural quality of the models. Sequence identities of 40% to 65% between respective template and target sequences furthermore suggest that the global folds are conserved. Note that we generally refer to the simulated systems as “TMD” here, although the actual TM region is prepended by 9–11 (8) residues of the linker to the calf-2 (β-tail) domain at the N-terminal end of the α (β) subunit, and appended by 5 (6) residues of the respective cytosolic domains at the C-terminal ends (Fig. 1b). For clarity, we furthermore only refer to the sequence numbering of integrin α_IIb_β_3_ below (Fig. 1b).

Using the three TMD structures as starting structures, we performed three independent MD simulations of 1 μs length for each system, yielding in total 9 μs of simulation time. The structural variability was assessed in terms of root mean square deviations (RMSD) after a mass-weighted superimpositioning onto the respective starting structure. The RMSD of all C_α_ atoms, including those not embedded in the membrane, raises to values of ~7–10 Å. However, if only the TM region is considered, the RMSD amounts to ~2–4 Å (Fig. 1c). This data indicates that the structural integrity of the two TM helices remains intact throughout the MD simulations, whereas the TMD ends fray at about nine residues at either terminus of either helix. The finding that the overall configuration of the TMD remains intact during our simulations is congruent with a slow dissociation rate found for α_IIb_β_3_ TMD^44^. In agreement with our findings, NMR spectroscopy revealed a dynamically unstructured α_IIb_ linker^45^. The convergence of the internal motions between independent MD simulations was assessed following ref.^46^. In short, the overlap of histograms of principal component (PC) projections obtained in a pair-wise manner from each simulation for a given TMD system are looked at as a function of time (Fig. S2). The results reveal that the first three PCs are relatively well-converged after ~400 ns for α_IIb_β_3_ TMD, whereas for α_v_β_3_ and α_5_β_1_ TMDs it takes ~800 ns to reach a comparable level of convergence.

To conclude, RMSD values of membrane-embedded TMD parts generally below 4 Å reveal that both the secondary structure and overall configuration of the two TM helices remain intact throughout the MD simulations of all three TMDs. The convergence behavior of PC projections suggests larger and/or slower internal motions in α_v_β_3_ and α_5_β_1_ TMDs than α_IIb_β_3_ TMD.

### Residue-wise analysis of contacts and mobility in the TMD interfaces

Next, we examined differences in the TMD topology of each system in terms of changes in the number of contacts present in the starting structure (“native contacts”) and those formed over the MD simulation time (“non-native contacts”). A contact is considered formed between the α and β subunits if any two atoms of two residues come closer than 7 Å^47^. First, for globally comparing the three systems, we computed the average number of overall contacts. The total number of native contacts already present in the NMR structure of α_IIb_β_3_ TMD (PDB ID 2K9J) is almost equal to that of the initial structure of α_v_β_3_ TMD (6217 *versus* 6244), and only ~2% smaller than that of the initial structure of α_5_β_1_ TMD (6344). In contrast, compared to α_IIb_β_3_, both α_v_β_3_ and α_5_β_1_ reveal a highly significant reduction of overall contacts by ~10% (*p* < 0.0001) (Fig. S3) during the MD simulations. Second, from a list of residues conserved across the three α subunits and the contact map information, we extracted those residues accounting for the native and non-native contacts at the OMC and IMC interface between α and β subunit (Table S1). As to the former, compared to α_IIb_β_3_ TMD, the overall average number of contacts is ~20% smaller in the α_v_β_3_ TMD and ~40% smaller in the α_5_β_1_ TMD (Fig. 1d). Both differences are highly significant (*p* < 0.0001). As to the latter, compared to α_IIb_β_3_ TMD, both α_v_β_3_ and α_5_β_1_ TMDs reveal a significant reduction of overall contacts by ~10% (Fig. 1e) (*p* ≈ 0.003 and 0.006, respectively).

Furthermore, we computed residue-wise root mean square fluctuations (RMSF) as a measure of atomic mobility to identify local differences in the structural dynamics of the three TMD complexes, averaged over the respective three simulations. For both the α and β subunit, the α_IIb_β_3_ TMD shows less pronounced residue motions, with the RMSF values of 60 out of 89 residues (~67%) being lower than those of α_v_β_3_ and α_5_β_1_ (Table S2). To conclude, these results demonstrate on a per-residue level that the α_IIb_β_3_ TMD forms generally more contacts across the TMD interface and shows less mobile residues in the TMD.

### Subunit-specific differences in OMC and IMC distances

Both the OMC and IMC are considered necessary to maintain integrin in the low affinity state^2^ (Fig. 1a). However, particularly in the OMC, each TMD has subunit-specific amino acids (Fig. 1b) that may be responsible for the above observed differences. The *d*_OMC_ (distance computed between the centers of mass (COM) of the C_α_ atoms of the G*XXX*G motif (G972-G976) on the α_IIb_ subunit and V700-I704 on the β_3_ subunit) is 9.3 Å in the NMR structure of α_IIb_β_3_ TMD (PDB ID 2K9J) and 7.3 Å in a structure based on Cys cross-linking results^48^. The distance *d*_IMC_ computed as the minimal distance between the COMs of the aromatic rings of F992 or F993 on the α_IIb_ subunit and the aromatic ring of W715 on the β_3_ subunit is 4.0 Å in the NMR structure and 5.4 Å in the structure based on Cys cross-linking results.

**Figure 2:**
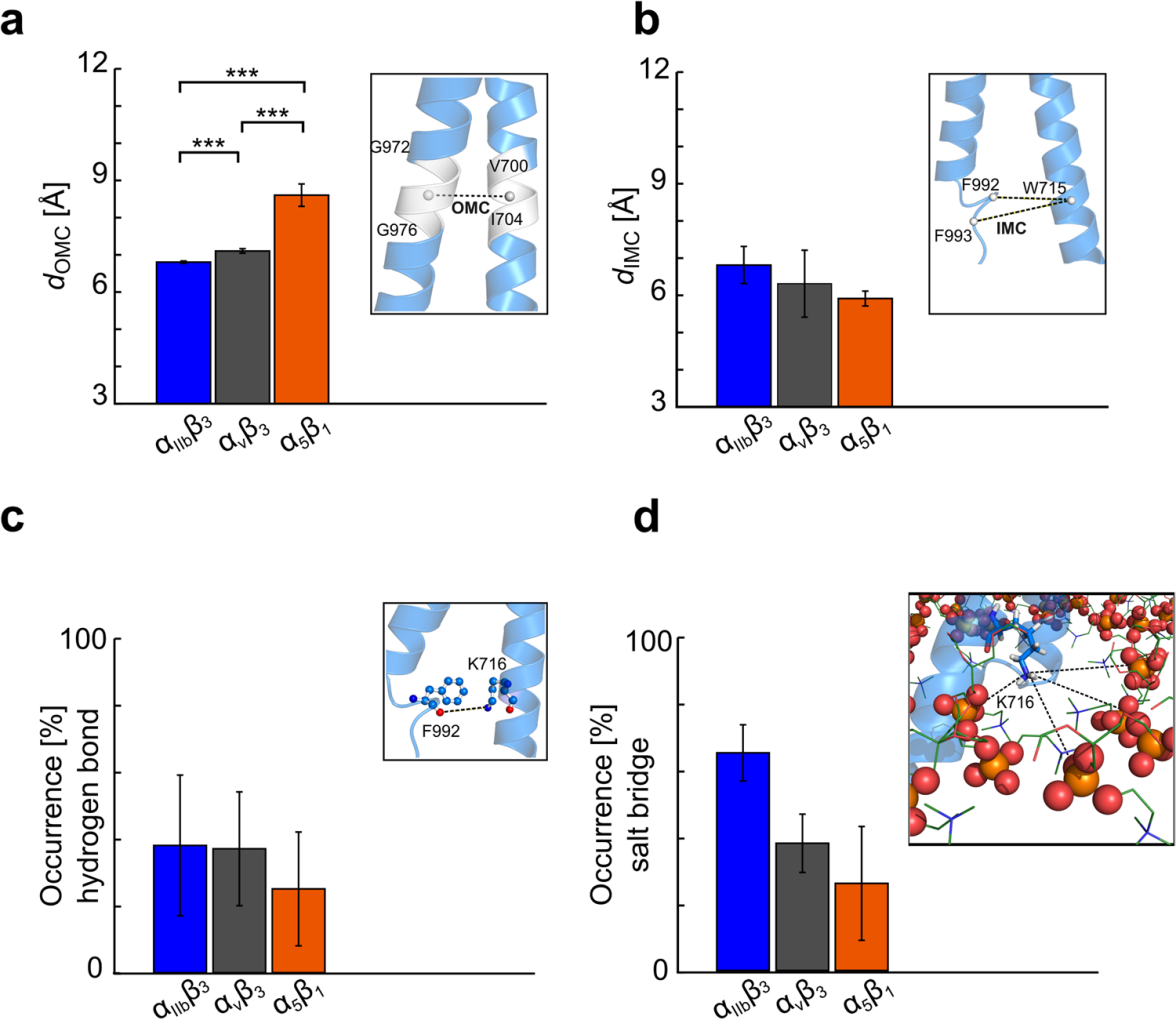
Differences between interdomain interactions in α_IIb_β_3,_ α_v_β_3,_ and α_5_β_1_ TMDs. (**a**,**b**) Histograms of the distances *d*_OMC_ and *d*_IMC_ (see main text for definition) averaged over three MD simulations. From left to right, the α_IIb_β_3_, α_v_β_3_, and α_5_β_1_ TMDs are displayed in blue, grey, and orange, respectively. Within each plot, a close-up view of the NMR structure of α_IIb_β_3_ TMD (PDB ID 2K9J) is colored in blue and indicates the analyzed distances (black dashed lines). White spheres indicate C_α_ atoms of the labeled amino acids. (**c**) Histogram of the mean relative occurrence of the hydrogen bond between K716_Nε_ and F992_O_ using a distance cutoff of 3.5 Å and an angle cutoff of 120°. Within the plot, a close-up view of the respective distance measured is shown (color code as in panel a). (**d**) Histogram of the mean relative occurrence of the salt bridge between K716_Nε_ and the oxygens of phospholipids head groups of the lower lipid leaflet applying a distance cutoff of 4 Å. Within the plot, a close-up view of the respective distance measured is shown, with the phospholipid head groups depicted as spheres. (**a**–**d**) Error bars show the SEM (eq. 5) and stars indicate the statistical difference (see Methods section for definition).

As to the MD simulations, first, the *d*_OMC_ averaged over three simulations is smaller by ~0.3 Å in the α_IIb_β_3_ TMD (~6.8 Å, SEM ≈ 0.03 Å) than in the α_v_β_3_ TMD (~7.1 Å, SEM ≈ 0.06 Å), and smaller by ~1.5 Å than in the α_5_β_1_ TMD (~8.4 Å, SEM ≈ 0.3 Å). All values are well in the range of inter-helical distances found for TM heterodimers containing an OMC-like structural motif^49^. The differences are highly significant in all cases (*p* < 0.0001) (Fig. 2a, Table S3). Hence, the OMC interface is most compact in α_IIb_β_3_ TMD, followed by α_v_β_3_ and α_5_β_1_ TMDs. Second, the *d*_IMC_ averaged over three simulations remains below 7 Å in all cases: α_IIb_β_3_ (~6.8 Å, SEM ≈ 0.5 Å), α_v_β_3_ (~6.3 Å, SEM ≈ 0.9 Å) and α_5_β_1_ TMDs (~5.9 Å, SEM ≈ 0.2 Å) (Fig. 2b, Table S3). The differences are not significant (*p* ≈ 0.6 for α_IIb_β_3_/α_v_β_3_, ≈ 0.05 for α_IIb_β_3_/α_5_β_1_, ≈ 0.5 for α_v_β_3_/α_5_β_1_), however. Hence, we conclude that the IMC interface is equally maintained over the course of the MD simulations.

### Subunit-specific interactions formed across the TMD interfaces

The IMC interface is adjacent to the membrane-proximal region, which is believed to be mainly stabilized by a salt bridge formed between α_IIb_-R995 and β_3_-D723^2^. First, we computed the minimal distance between the atoms R995_Nη1/Nη2_ and D723_Oδ1/Oδ2_ for each system (Table 1). As next to R995 and D723, respectively, R997 is present on the α subunit and E726 on the β subunit, we also analyzed the minimal distances between atoms R995_Nη1/Nη2_ and E726_Oε1/Oε2_, R997_Nη1/Nη2_ and D723_Oδ1/Oδ2_, and R997_Nη1/Nη2_ and E726_Oε1/Oε2_ (Table 1; α_5_β_1_ integrin contains a Leu at position 997 such that the last two distances were not evaluated there). Then, to assess the frequency of occurrence of formed salt bridges, we applied a cutoff of 4 Å to the computed minimal distances, according to a previous study^50^ (Table 1): 1) the R995-D723 salt bridge has the highest occupancy in the α_IIb_β_3_ TMD (~58%), followed by the α_v_β_3_ (~37%) and α_5_β_1_ TMDs (~39%). The differences between α_IIb_β_3_ TMD and either α_v_β_3_ or α_5_β_1_ TMDs are not statistically significant (*p* ≈ 0.3 for α_IIb_β_3_/α_v_β_3_, α_IIb_β_3_/α_5_β_1_, and ≈ 0.9 for α_v_β_3_/α_5_β_1_); 2) the R995-E726 salt bridge (the only alternative interaction that can be formed in α_5_β_1_) has the highest occupancy in the α_5_β_1_ TMD (~42%), followed by the α_IIb_β_3_ (~33%) and α_v_β_3_ TMDs (~39%). The differences are not significant (*p* ≈ 0.8 for α_IIb_β_3_/α_v_β_3_, α_IIb_β_3_/α_5_β_1_, and ≈ 0.9 for α_v_β_3_/α_5_β_1_); 3) the R997-D723 salt bridge is formed in both the α_IIb_β_3_ (~47%) and α_v_β_3_ TMDs (~48%) to a very similar extent (the difference is not significant (*p* ≈ 0.9)); 4) the R997-E726 salt bridge is formed to a lower extent in the α_IIb_β_3_ (~17%) and α_v_β_3_ TMDs (~31%), and the difference is not significant (*p* ≈ 0.4). To conclude, our results, in agreement with ref.^24^, indicate that R995-D723 is not the only salt bridge that is formed across the TMD interface. In agreement with ref.^51^, the R995-D723 salt bridge dissociates intermittently, as indicated by occupancies ≪100%. However, the MD simulations reveal that, among all four possible salt bridges that can form in the membrane-proximal region, R995-D723 is the most prevalent interaction, followed by R995-E726, and R997-E726 is the least prevalent one.

**Table 1:** Frequency of occurrence of the up to four salt bridges in the membrane-proximal region for each TMD system^[a]^.

Salt bridge	α_IIb_β_3_ TMD			α_v_β_3_ TMD			α_5_β_1_ TMD		
	**SimI**	**SimII**	**SimIII**	**SimI**	**SimII**	**SimIII**	**SimI**	**SimII**	**SimIII**
R995-D723	47	92	35	57	8	46	14	66	38
	58 ± 6.7^[b]^			37 ± 14.8^[b]^			39 ± 15.0^[b]^		
R995-E726	47	50	2	70	29	18	24	78	24
	33 ± 15.5^[b]^			39 ± 18.8^[b]^			42 ± 18.0^[b]^		
R997-D723	23	95	23	84	52	7	—^[c]^	—^[c]^	—^[c]^
	47 ± 24.0^[b]^			48 ± 22.3^[b]^			—^[c]^		
R997-E726	10	39	2	10	31	51	—^[c]^	—^[c]^	—^[c]^
	17 ± 11.2^[b]^			31 ± 11.8^[b]^			—^[c]^		

^[a]^In %.

^[b]^Mean values and SEM (eq. 6), calculated over three MD simulations.

^[c]^Interactions that cannot be formed in the TMD of integrin α_5_β_1_.

### Subunit-specific contribution of K716 to the TMD stability

Finally, we investigated the behavior of K716, monitoring where the residue preferentially places its side chain, i.e., either as part of a hydrogen bond with F992 or by orienting the positive charge of N_ε_ towards the negatively charged head groups of the lipid bilayer^38^. In the NMR structure of α_IIb_β_3_ TMD (PDB ID 2K9J), the distance between N_ε_ of K716 and the carbonyl oxygen of F992 is 6.2 Å; in the structure based on Cys cross-linking results, it is 2.9 Å. During the three simulations of each system, the occupancy of the K716-F992 hydrogen bond was investigated applying a distance cutoff of 3.5 Å and an angle cutoff of 120°. On average, the hydrogen bond is formed in a similar manner in the α_IIb_β_3_ (~38%) and α_v_β_3_ TMDs (~37%), and to a lower extent in the α_5_β_1_ TMD (~25%) (Fig. 2c, Table S4). The differences between α_IIb_β_3_ or α_v_β_3_ TMDs with respect to α_5_β_1_ TMD result in *p* ≈ 0.6, respectively.

To evaluate the presence of electrostatic interactions between the K716 side chain and the head groups of lipids, first, we computed the minimal distance between N_ε_ of K716 and the nearest O atom of the phospholipid head groups from the lower lipid leaflet (*d*_snorkeling_). Then, we applied a 4 Å cutoff to *d*_snorkeling_ and calculated the frequency of occurrence of a salt bridge. On average, the salt bridge is mainly formed in the α_IIb_β_3_ TMD (~65%), followed by α_v_β_3_ TMD (~38%), and to a lower extent in the α_5_β_1_ TMD (~26%) (Fig. 2d, Table S5). The differences between α_IIb_β_3_ TMD with respect to α_v_β_3_ or α_5_β_1_ TMDs result in *p* ≈ 0.08 and ≈ 0.1, while the difference between α_v_β_3_ and α_5_β_1_ TMDs results in *p* ≈ 0.5. To conclude, the K716 sidechain is engaged in either a hydrogen bond across the TMD or a salt bridge with phospholipid head groups in the α_IIb_β_3_ TMD (~100%), followed by α_v_β_3_ (~75%) and α_5_β_1_ (~50%) TMDs, with the salt bridge being the more prevalent interaction in the case of α_IIb_β_3_ TMD.

### Configurational free energies of TM helix association

**Figure 3:**
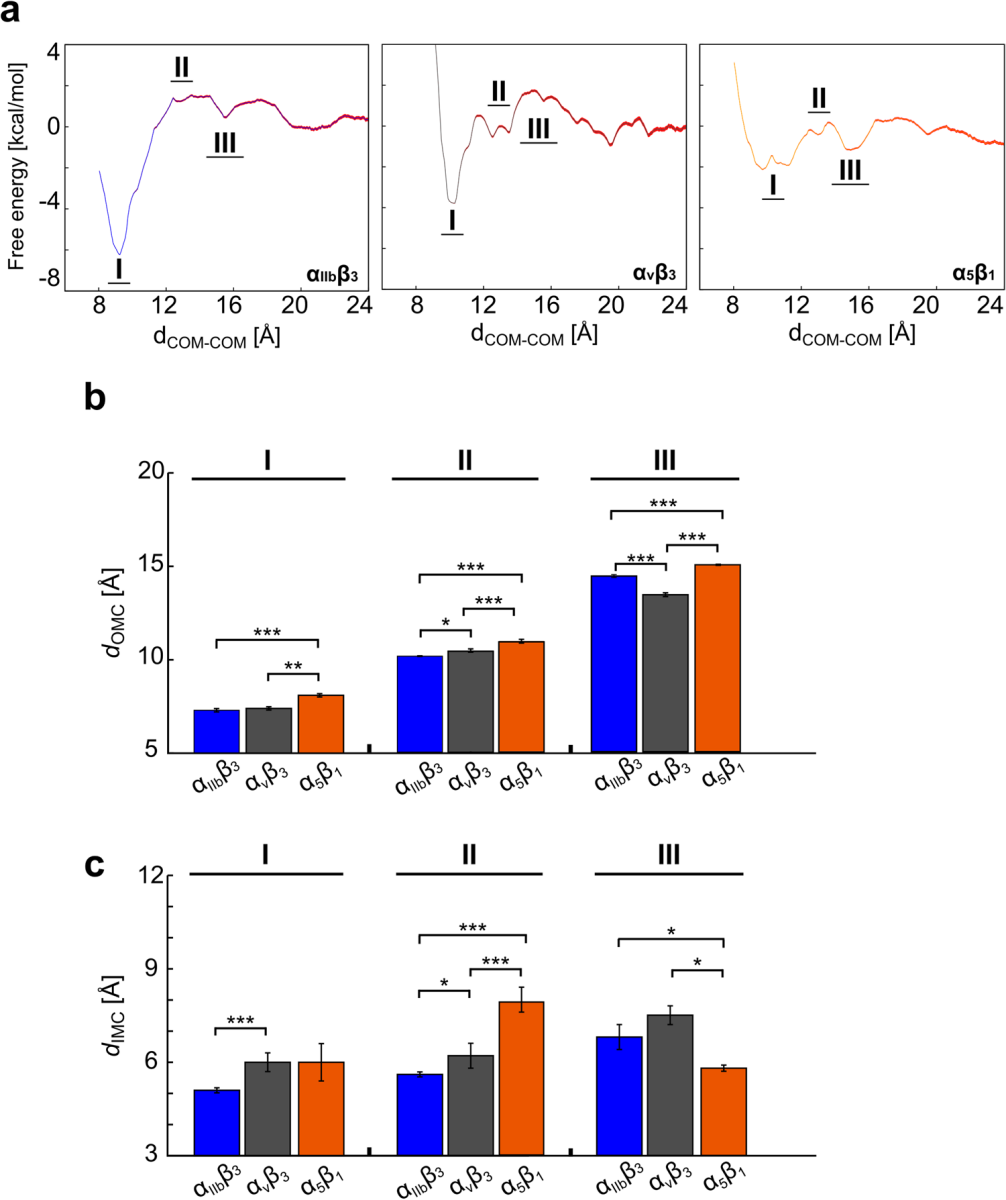
Potential of mean force of TM helix association of α_IIb_β_3_, α_v_β_3_, and α_5_β_1_ TMDs and differences at the OMC/IMC interface with increasing *d*_COM-COM_. (**a**) Configurational free energies as a function of the *d*_COM-COM_ used as a reaction coordinate for α_IIb_β_3_ (left panel), α_v_β_3_ (middle panel), and α_5_β_1_ (right panel) TMDs. Roman numbers indicate free energy minima. Statistical errors, calculated using bootstrap analysis, are displayed as red shaded curves added to the PMF profiles. The PMF values at *d*_COM-COM_ = 20 Å were set to zero. (**b**,**c**) Histograms of the averaged *d*_OMC_ (B) and *d*_IMC_ (C) across umbrella sampling windows linked to free energy minima I–III observed in panel (A) (see also Table S7), using reweighted (“unbiased”) TMD configurations. Error bars denote the SEM (eq. 5) and stars indicate the statistical difference (see Methods section for definition).

The above analyses strongly suggest that the TMDs of the integrin isoforms differ in their potential to associate. To corroborate these findings in an independent manner, we computed the configurational free energy (potential of mean force, PMF) of TM helix association using the distance between COMs of the sections of the α and β subunits embedded in the membrane as a reaction coordinate (referred to as *d*_COM-COM_). The PMFs were computed using umbrella sampling^52^ along a pathway from the bound subunits to subunits where any pair of atoms between the two subunits is at least 10 Å apart, and WHAM^53^ post-processing. The PMF profiles were obtained employing 16 biased MD simulations of 200 ns length for reaction coordinate values of *d*_COM-COM_ = 8 Å to 20 Å, and four biased MD simulations of 70 ns length for reaction coordinate values of *d*_COM-COM_ = 21 Å to 24 Å. Together, this sums up to a total of ~ 3.5 μs simulation time. Approximately Gaussian-shaped frequency distributions were obtained for each reference point along the reaction coordinates, with all such distributions well overlapping (Fig. S4). These are prerequisites for the successful application of WHAM to extract a PMF from these distributions^53^. Repeating the computations of the PMFs for the range of *d*_COM-COM_ = 8 Å to 20 Å for parts of the simulation time demonstrates that, for all three systems, the PMFs are converged after at most 160 ns of simulation time per window (maximal difference between two PMFs: 0.2 kcal mol^−1^) (Fig. S5). The same procedure was repeated for the PMFs from *d*_COM-COM_ = 20 Å to 24 Å (fully dissociated state), demonstrating that these PMFs are converged after at most 50 ns of simulation time per window (Fig. S5). For comparison, the PMF values at *d*_COM-COM_ = 20 Å were set to zero in all three cases (Fig. 3a).

The global minima (*d*_COM-COM_ ≈ 9 Å) are in all three cases close to the initial distance (10 Å resp. 12.6 Å) calculated from the NMR structure of the α_IIb_β_3_ TMD (PDB ID 2K9J) or the structure based on Cys cross-linking results, and the general shapes of the PMFs are comparable, with a rising free energy with increasing reaction coordinate values and rather flat PMFs beyond *d*_COM-COM_ ≈ 20 Å. Furthermore, in going from the global minima to *d*_COM-COM_ ≈ 20 Å, two local minima are passed (marked by roman numbers II and III in Fig. 3a). These minima are located at *d*_COM-COM_ ≈ 12 to 13 Å and *d*_COM-COM_ ≈ 15 Å in all three systems.

However, the PMFs also show pronounced differences. First, the global minima show values of −6.5, −3.8, and −2.1 kcal mol^−1^ for the α_IIb_β_3_, α_v_β_3_, and α_5_β_1_ TMDs, respectively, demonstrating the largest tendency for the TM helices to associate in the case of α_IIb_β_3_, and the lowest in the case of α_5_β_1_. For computing association free energies Δ*G* from the PMFs (eqs 1–3), we, first, assessed to what extent the dissociated TMD helices of α_IIb_ and β_3_ sample the accessible configuration space on the simulated time scales. This yielded values for ||*Ω*||, a factor describing the restriction of the configurational space of the monomers upon dimer formation (eq. 4), of 0.05 to 0.10 (Table 2). Compared to the value of the overall accessible space of (2π)^2^, these values indicate that the dissociated TMD helices sample only a fraction of the overall accessible space. Hence, we followed a procedure by Johnston *et al*.^54^ to compute dimerization and mole fraction dimerization constants *K*_*a*_ and *K*_X_, respectively, (eqs 1,2) and from there Δ*G* (eq. 3) (Table 2). The results indicate that α_IIb_β_3_ TMD is the most stable system (Δ*G* = −3.8 kcal mol^−1^), followed by α_v_β_3_ and α_5_β_1_ TMDs (Δ*G* = −0.8 kcal mol^−1^ and 0.5 kcal mol^−1^, respectively). Note that the exact value of the dimerization distance *D* (eq. 1; here evaluated between 8 and 12 Å) has a low impact on the results (see Table S6 for Δ*G* values evaluated between 8 and 14 Å), because the integrals reach plateaus after ~10 Å^55^. Second, the TM association requires to pass configurational free energy barriers that are similar for α_IIb_β_3_ and α_5_β_1_ TMDs (~1.5 kcal mol^−1^, respectively), whereas a larger barrier occurs in the case of α_v_β_3_ TMD (~3 kcal mol^−1^).

**Table 2:** Thermodynamic quantities for each TMD system^[a]^.

System	α_IIb_β_3_ TMD	α_v_β_3_ TMD	α_5_β_1_ TMD
||*Ω*||^[b]^	0.07	0.05	0.1
*K*_*a*_^[c]^	38009.8	280.9	27.7
*K*_X_	543.0	4.0	0.4
Δ*G*^[d]^	−3.8	−0.8	0.5

^[a]^The integral in eq. 1 was evaluated at *D* = 12 Å.

^[b]^In radians.

^[c]^In Å^2^.

^[d]^In kcal mol^−1^.

To conclude, the PMFs of TM helix association and computed association free energies show overall similar shapes but reveal that the α_IIb_β_3_ TMD has the strongest tendency to associate, and the PMF of the α_5_β_1_ TMD shows the smallest configurational free energy changes.

### Distinct order of TMD clasp formation and differences in the persistence of IMC and OMC with increasing helix-helix distance

To further investigate differences between the integrin isoforms in more detail, averaged *d*_OMC_ and *d*_IMC_ were computed on the reweighted (“unbiased”) configurations from umbrella sampling (reweighting done according to ref.^56^) (Fig. S6) for free energy minima I – II, respectively (Fig. 3b,c; Table S7).

At the global minimum I, the *d*_OMC_, averaged over windows 3–5, is very similar in the α_IIb_β_3_ and α_v_β_3_ TMDs (~7.3 Å, ~7.4 Å, SEM ≈ 0.09 Å), and smaller by ~0.8 Å than in the α_5_β_1_ TMD (~8.1 Å, SEM ≈ 0.09 Å). The differences between α_IIb_β_3_ or α_v_β_3_ TMDs *versus* α_5_β_1_ TMD are (highly) significant (*p* < 0.0001, and *p* ≈ 0.0003). The *d*_IMC_ is smaller by ~0.9 Å in the α_IIb_β_3_ TMD (~5.1 Å, SEM ≈ 0.08 Å) than in the α_v_β_3_ and α_5_β_1_ TMDs (~6.0 Å, SEM ≈0.3 Å and ≈ 0.6 Å, respectively). The difference between α_IIb_β_3_ and α_v_β_3_ TMDs is highly significant (*p* < 0.0001), while for that between α_IIb_β_3_ and α_5_β_1_ TMDs, *p* ≈ 0.06 results. Hence, both the OMC and IMC are conserved, with the clasps being more compact in the α_IIb_β_3_ TMD, followed by α_v_β_3_ and α_5_β_1_ TMDs.

At minimum II, the *d*_OMC_, averaged over windows 8–9, is slightly smaller in the α_IIb_β_3_TMD (~10.1 Å, SEM ≈ 0.03 Å) than in the α_v_β_3_ TMD (~10.4 Å, SEM ≈ 0.09 Å), and smaller by ~0.8 Å than in the α_5_β_1_ TMD (~10.9 Å, SEM ≈ 0.1 Å). The difference between α_IIb_β_3_ and α_v_β_3_ TMDs is significant (*p* ≈ 0.04) and highly significant in the other cases (*p* < 0.0001). The *d*_IMC_ is smaller by ~0.6 Å in the α_IIb_β_3_ TMD (~5.6 Å, SEM ≈ 0.08 Å) than in the α_v_β_3_ TMD (~6.2 Å, SEM ≈ 0.4 Å), and by ~2.4 Å than in the α_5_β_1_ TMD (~8.0 Å, SEM ≈ 0.4 Å). The difference between α_IIb_β_3_ and α_v_β_3_ TMDs is significant (*p* ≈ 0.03 Å), and highly significant in the other cases (*p* < 0.0001). Hence, the interface at the OMC is less compact than before and starts to disintegrate (*d*_OMC_ > 10 Å). In contrast, the IMC packing is conserved, similar to as before, but the interface is tighter in α_IIb_β_3_ TMD than in α_v_β_3_ and α_5_β_1_ TMDs.

Finally, at minimum III, *d*_OMC_ and *d*_IMC_, averaged over windows 11–12, reveal that the OMC packing is largely lost (distances > 13 Å), while the IMC packing is still conserved. The *d*_IMC_ is ~0.8 Å smaller in the α_IIb_β_3_ TMD (~6.8 Å, SEM ≈ 0.4 Å) than in the α_v_β_3_ TMD (~7.5 Å, SEM ≈ 0.3 Å), and ~1 Å larger than in the α_5_β_1_ TMD (~5.8 Å, SEM ≈ 0.1 Å). The difference between α_IIb_β_3_ and α_v_β_3_ TMDs is not significant (*p* ≈ 0.2), but both differences *versus* α_5_β_1_ TMD are significant (*p* ≈ 0.005).

To conclude, our results suggest for all investigated integrin isoforms that helix association in the TMDs proceeds first via IMC formation, and that OMC formation then reinforces the coiled-coil conformation. The reverse order is suggested to occur upon helix dissociation. However, pronounced differences among the three TMDs as to the conservation of OMC/IMC packing with increasing *d*_COM-COM_ became obvious, with α_IIb_β_3_ TMD showing the most persistent clasps.

## Discussion

In this study, we have shown by molecular simulations at the atomistic level that the TMD of integrin α_IIb_β_3_ is most stably associated compared to that of α_v_β_3_ and α_5_β_1_, and that this difference is related to differences in particular interactions across the TMDs, notably in the OMC. We furthermore identified an “OMC before IMC” order of clasp disintegration upon TMD dissociation as a uniform property of all three investigated TMDs. Our study was motivated by the considerable evidence that the TMD separate upon integrin activation^7,11,13,14,17,57–60^ and so are expected to influence conformational properties of integrins, which, in turn, have been correlated to integrin adhesiveness and affinity^26,61–66^. Furthermore, while the overall structural organization and activation mechanism of integrins appears largely conserved^48,62,66–68^, subunit-specific differences in the activation sensitivity had been reported^26,27,29^. Accordingly, here, we comparatively assessed the TMD of α_5_β_1_, a physiologically relevant^69,70^ member of the β_1_ integrin subfamily that is considered basally active^26^ and TMDs of α_IIb_β_3_ and α_v_β_3_, physiologically relevant^71,72^ members of the β_3_ integrin subfamily that is considered basally inactive^26^, although a cell-specific influence on the activation sensitivity has been reported for integrin α_v_β_3_^29^.

That α_IIb_β_3_ TMD is most stably associated compared to that of α_v_β_3_ and α_5_β_1_ has been demonstrated in two independent ways. First, we performed unbiased microsecond-long MD simulations at the atomistic level in explicit solvent and an explicit lipid bilayer on the three TMDs, likely the currently most accurate way to explore structure and dynamics of transmembrane proteins^73^. The length of our MD simulations surpasses comparable previous ones on integrin TMDs by at least one order of magnitude^51,74,75^. We performed triplicate MD simulations for each system, which allows probing for the influence of the starting conditions and determining the significance of the computed results by statistical testing and rigorous error estimation^46^. As to the latter, we paid close attention to only consider uncorrelated instances for SEM calculations (eqs 5 and 6). The assessment of convergence of internal motions between independent MD simulations revealed for motions of the TMDs described by the first three PCs that they are relatively well-converged on the timescale of the simulations. For the MD simulations, we used established parameterizations for the solvent^76^, lipids^77^, and proteins^78^; the latter, we had also applied successfully in other integrin simulations^79–82^, although we note that more recent protein force fields have become available^83,84^. Yet, the impact of force field deficiencies on our results is expected to be small due to cancellation of errors when comparatively assessing the TMDs. While for α_IIb_β_3_ TMD an experimental structure was available for system setup^15^, structures generated by homology modeling were used for α_v_β_3_ and α_5_β_1_ TMDs. Still, with sequence identities of 40 to 65%, C_α_-RMSD values to the native structure below 1 Å and, hence, close to experimental uncertainty can be expected for transmembrane regions^85^. The quality of the modeled starting structures is indirectly supported by the fact that for all three systems, very similar magnitudes of structural deviations along the MD trajectories were found (Fig. 1c). The simulated protein sequences contain a linker region at the N-terminal ends and up to six residues of the cytoplasmic domains at the C-terminal ends, in addition to the TM helices. For β_1_ and β_3_, the linker region is almost conserved (Fig. 1b), and the linker of α_IIb_ has been shown to be dynamically unstructured^45^. Together with crystallographic studies of inactive ectodomains that were unable to obtain structural information on these linkers, indicating their high flexibility^48,86,87^, we thus do not expect these linker regions to influence the TMD association differentially. In contrast, for the cytoplasmic membrane-proximal region, an influence on maintaining integrin inactivity has been suggested^36^ (see also below). Of particular relevance is the choice of lipid type, as it has been shown that annular anionic lipids can stabilize α_IIb_β_3_ TMD^51^. Therefore, our lipid bilayer consisted of zwitterionic 1,2-dioleoyl-*sn*-glycero-3-phosphocholine (DOPC) lipids, which were shown to interfere much less with inter-TM helix interactions^51^.

As a second, independent means to investigate the energetics of TMD association and because we cannot expect to observe a dissociation of the TMDs on the time scale of our MD simulations^44^, we performed biased MD simulations at the atomistic level followed by PMF computations, using established protocols successfully applied previously by us^50,88,89^ and *d*_COM-COM_ as an intuitive reaction coordinate previously applied on similar systems^6,90^. To our knowledge, so far, the association energetics of integrin TMDs, by computational means, has only been investigated by coarse-grained MD simulations followed by PMF computations^6,90^. By repeating the PMF computations for parts of the biased simulations, we demonstrated that the PMFs are converged with respect to the overall simulation time per sampling window (Fig. S5). Still, even with sampling times of up to 200 ns per sampling window, pronounced helix tilting or even helix rotation around an axis perpendicular to the helix axis, once the helices are separated by a large enough distance, cannot be expected. Likewise, such sampling times may not be sufficient to yield sampled helix-helix configurations that are completely unbiased from the respective starting structures. While these potential issues may be expected to disfavor separated helix-helix configurations with respect to associated TMD, their impact on our results is expected to be small due to cancellation of errors when comparatively assessing the respective TMD. Comparing quantitative results from the PMFs (Fig. 3a) and subsequent association free energy calculations^54^ (Table 2) to experimental data lends remarkable support to the quality of the setup, parameterization, and execution of our simulations: (I) For α_IIb_β_3_ TMD, association free energies of −4.33 and −4.84 kcal mol^−1^ have been determined in 1-palmitoyl-2-oleoyl-*sn*-glycero-3-phosphocholine (POPC) lipids by NMR spectroscopy and calorimetry^44,51^, and our computed Δ*G* of −3.8 kcal mol^−1^ (Table 2) is within chemical accuracy of these results; (II) for α_5_β_1_, to our knowledge, the energetics of TMD association has not been explicitly probed experimentally. However, from comparing conformational equilibria between the extended-closed and extended-open states for full-length integrin α_5_β_1_ *versus* the α_5_β_1_ ectodomain, one may infer that the associated and dissociated states of the TMDs and cytoplasmic domains are almost isoenergetic^26^, which is in very good agreement with a computed Δ*G* of 0.5 kcal mol^−1^ (Table 2); (III) obtaining a barrier height pertinent to kinetics *via* a PMF has been debated^91^. Still, when inserting the configurational free energy barrier for α_IIb_β_3_ TMD association of ~1.5 kcal mol^−1^ (Fig. 3a) as Δ*G*^‡^ in the transition state theory equation *k* = *k*^‡^ exp(−Δ*G*^‡^/*RT*)^92^ and approximating *k*^‡^ with a collision frequency of a transmembrane protein of 10^5^ to 10^6^ s^−1^ ^93^, a rate *k* of ~8 * 10^3^ to ~8 * 10^4^ s^−1^ is obtained as a coarse upper bound^94^, in good to fair agreement with an association rate of α_IIb_β_3_ TMD in phospholipid bicelles of 4.5 * 10^3^ s^−1^ found by NMR spectroscopy^44^. As to probing the internal consistency of our results, the shallow contact minimum found in the PMF for α_5_β_1_ TMD and the apparent lack of pronounced barriers towards the dissociated state suggest that in MD simulations that are long enough, the two helices should (start to) come apart. In fact, such a tendency is found as *d*_COM-COM_ in the unbiased MD simulations of α_5_β_1_ TMD is ~1 and 0.6 Å larger than for α_IIb_β_3_ and α_V_β_3_ TMDs (Fig. S7, Table S8). Likewise, internal consistency can be probed by analyzing structural parameters of configurations from unbiased MD simulations and reweighted (“unbiased”) configurations from umbrella sampling simulations. This reveals qualitatively similar results for subtype-specific differences in the distances characterizing OMC formation (*d*_OMC_; Fig. 2a *versus* Fig. 3b, part I), and likewise similar results for α_v_β_3_ and α_5_β_1_ TMDs concerning the distance characterizing IMC formation (*d*_IMC_; Fig. 2b *versus* Fig. 3c, part I), although the results for α_IIb_β_3_ deviate in this case. The latter discrepancy may be explained in that distances in Fig. 2b where directly computed from the (tightly associated) α_IIb_β_3_ TMD configurations in the unbiased MD simulations, while three umbrella sampling windows were evaluated for distances in Fig. 3c.

The PMF and association free energy calculations clearly revealed that α_IIb_β_3_ TMD is most stably associated, followed by α_v_β_3_ TMD and α_5_β_1_ TMD (Table 2). It had been previously suggested by experiment^13,39^ and computations^49,95^ that the two structural motifs OMC and IMC are responsible for the correct TMD packing but if, and how, sequence variations there (Fig. 1b), especially in the OMC, lead to differential TMD association has remained largely elusive. Our unbiased MD simulations reveal that the most stable association of α_IIb_β_3_ TMD is paralleled by this TMD forming more contacts in general across the whole TMD interface (Fig. S3) as well as particularly in the OMC interface (Fig. 1d); also, this TMD shows the most compact OMC (Fig. 2a; Fig. 3b in part I). Although differences in the prevalence of single hydrogen bonds or salt bridges among the three TMDs were not significant, our unbiased MD simulations still suggest a trend, according to which the importance of the membrane-proximal region for TMD association is confirmed, particularly of the R995-D723 salt bridge^24,39,51^, as this interaction is also most prevalent in α_IIb_β_3_ TMD (>20%, Table 1). Our results may also provide an explanation as to why, in *in vivo* experiments on transgenic mice carrying a point mutation of the respective D723 of the β_1_ subunit, a normal integrin function was found^32^: In α_5_β_1_ TMD, the R995-E726 salt bridge is more prevalent than the R995-D723 one. Other questions relate to the role of β_3_-K716 as a key determinant for the stability at the IMC interface^96^ and whether the proposed stabilization arises from an engagement of K716 with the surroundings lipid molecules^96^ or by forming a hydrogen bond with F992^17^. Our MD simulations revealed that K716 occurs most frequently in either a hydrogen bond across the TMD or a salt bridge with phospholipid head groups in α_IIb_β_3_ TMD, followed by α_v_β_3_ and α_5_β_1_ TMDs, consistent with the notion that K716 is important for integrin function in α_IIb_β_3_, but not in α_5_β_1_^25^.

Although more work would be required to establish unequivocally a link between differences in K716/phospholipid head group interactions and differences in TMD association free energies, we do not find it unlikely that this link exists. This link would then stress that (differences in) TMD association may be governed by additional factors besides sequence differences, including interactions to annular lipids, as previously demonstrated for the α_IIb_β_3_ TMD^51^, or membrane tension^97^. Along these lines, recent work on integrin α_5_β_1_ suggested the hypothesis that the non-ligand binding leg domains and *N*-glycans may have previously unappreciated roles in regulating integrin conformations^26^. Hence, while considering a subsystem such as the TMD in this study has the benefit of yielding detailed answers under well-defined conditions, at the same time, it leads to the limitation that effects from other parts of the system are not accounted for.

To our knowledge, a novel aspect resulting from our study with respect to the question what governs integrin adhesiveness and affinity in relation to conformational changes is the finding that the two clasps disintegrate in the order “OMC before IMC” upon TMD dissociation. This finding leads to the relevant prediction that the closed state of integrins might not be single but rather comprising several microstates that vary in the extent of TMD association, e.g., with the TMD associated at both OMC *and* IMC, or with the TMD associated only at the IMC. A similar proposition was made for the extended state(s) of integrins based on the flexibility of integrin legs^26,67^. Our finding may also be related to, not yet fully understood, results on conformational free energies for intact integrin α_5_β_1_ that revealed that the presence of the TMD and cytoplasmic domains favors considerably the bent-closed over the extended-closed conformation^26^, when one considers that the bent-closed → extended-closed transition may already contain energetic contributions from a partial TMD dissociation in the OMC region. In that respect, computations as performed here may support valuable efforts of gaining affinity information for specific integrin conformational states^26^ providing access to the energetic contributions of defined subsystems. Finally, we find it striking to note that the order of association free energies of the TMDs (α_IIb_β_3_ ≪ α_v_β_3_ < α_5_β_1_ (Table 2)) parallels reports on the basal activity of these integrins (α_IIb_β_3_ ≪ α_v_β_3_ < α_5_β_1_)^26,27^. While it is not possible to establish a direct link between these two series from the comparative simulation studies on TMDs alone, our finding suggests, to our knowledge for the first time, that the sequence composition of the TM helices can have a decisive effect on free energies associated with distinct conformational states of different integrins. A likewise suggestion has been made regarding the strength of interactions between leg domains for integrins α_IIb_β_3_ and α_v_β_3_^29^. Notably, the magnitude of differences in association free energies across the investigated TMDs (~2.7 kcal mol^−1^; Table 2) is clearly in the range of observed changes in conformational free energies upon activation of full-length integrin α_5_β_1_ (~3.7 kcal mol^−1^)^26^, suggesting that TMD differences can indeed significantly impact overall conformational integrin energetics.

In summary, α_IIb_β_3_ TMD is most stably associated compared to that of α_v_β_3_ and α_5_β_1_, which is related to differences in particular interactions across the TMDs, notably in the OMC. The order of TMD association stability is paralleled by the basal activity of these integrins, which suggests that TMD differences can have a decisive effect on conformational free energies of integrin states. The “OMC before IMC” order of clasp disintegration upon TMD dissociation uniformly identified for all three investigated TMDs suggests that the closed state of integrins might not be single but rather comprising several microstates that vary in the extent of TMD association.

## Methods

### Generation of starting structures

The starting structure for MD simulations of the α_IIb_β_3_ TMD was obtained from the coordinates of the NMR structure (PDB ID 2K9J) available in the RCSB Protein Data Bank^98^. The starting structures for MD simulations of the α_v_β_3_ and α_5_β_1_ TMDs were generated by homology modeling. The homology models were generated using MODELLER v9.9^99^, and the NMR structure of the α_IIb_β_3_ TMD was used as a template. In the case of α_v_β_3_ integrin, only the α_v_ sequence was modeled based on an alignment with the α_IIb_ TMD with a sequence identity of 45%. In the case of the α_5_β_1_ TMD, the α_5_ and β_1_ sequences are 40% and 65% identical to those of α_IIb_β_3_ TMD, respectively. The quality of the models was assessed by the QMEANBrane scoring function available in QMEAN server^42,43^. A global QMEAN score of 0.60, 0.64, and 0.67 was computed for the α_IIb_β_3_, α_v_β_3_, and α_5_β_1_ TMDs, respectively.

### Setup of simulation systems

The membrane builder tool available on the CHARMM-GUI website^100^ was used for embedding the TMDs in a pre-equilibrated bilayer of DOPC lipids^101^ using the replacement method^102^. The PPM web server^103^ was used to assess the correct orientation of the α_IIb_β_3_ TMD relative to the hydrocarbon core of the lipid bilayer. The required rectangular simulation box was generated by defining the number of lipids (88 and 85 for the upper and lower leaflet, respectively) and setting a value of 17 Å for the water layer above and below the protein. The total system size is ~60,000 atoms, including TIP3P water molecules^76^ and Cl^-^ counter ions.

### Molecular dynamics simulations

All MD simulations were performed using the AMBER 14 suite of programs^47^, the ff99SB force field for the proteins^78^, the Lipid14 force field^77^ for the lipids, and the TIP3P water model^76^. The particle mesh Ewald method^104^ was used to treat long-range electrostatic interactions, and bond lengths involving bonds to hydrogen atoms were constrained using the SHAKE algorithm^105^. The time step for integrating Newton’s equations of motion was 2 fs with a direct space, nonbonded cutoff of 8 Å. Initially, harmonic restraints with a force constant of 500 kcal mol^−1^ Å^−2^ were applied to all solute atoms during the first 250 steps of steepest descent and then reduced to 25.0 kcal mol^−1^ Å^−2^ for the second 2500 steps of conjugate gradient minimization and 10 kcal mol^−1^ Å^−2^ for the last 2500 steps of conjugate gradient minimization. MD simulations in the NVT (constant number of particles, volume, and temperature) ensemble were carried out for 50 ps, during which the system was heated from 100 to 300 K. Subsequent MD simulations in the NPT (constant number of particles, pressure, and temperature) ensemble were used for 150 ps to adjust the solvent density. In both steps, a force constant of 10 kcal mol^−1^ Å^−2^ was applied to all solute and lipid atoms. The production MD simulations of 9 μs length were performed with the GPU version of the program *pmemd*^106^ in the tensionless NPT ensemble using the anisotropic Berendsen barostat^107^ to control the pressure (coupling constant = 1 ps) and the Langevin thermostat^107^ to control the temperature (coupling constant = 1 ps), as suggested in ref.^77^.

### Potential of mean force computations

Profiles of the free energy of association of α_IIb_β_3_, α_v_β_3_, and α_5_β_1_ TMDs were constructed from umbrella sampling MD simulations^108,109^ in combination with the WHAM method^53^. As reaction coordination, the distance between the COMs of the TM segments embedded in the membrane was considered (C_α_ atoms of residues P996-V1015 and D718-I747, for the α_IIb_ and β_3_ subunits, respectively (equivalent ranges of residues were used for α_v_β_3_ and α_5_β_1_)). The initial distance computed from the NMR structure of the TMD of α_IIb_β_3_ integrin is 10.0 Å. To generate starting structures for umbrella sampling, the initial distance was reduced to 8 Å and increased to 11 Å in 0.5 Å steps, and from 11 Å increased to 24 Å in 1 Å steps. Each TMD configuration was inserted in a pre-equilibrated bilayer of DOPC lipids as described above. This resulted in a total of 20 initial systems per TMD. Each of the 20 windows of the three integrin systems was subjected to umbrella sampling simulations, carried out in the NPT ensemble for 200 ns each for *d*_COM-COM_ = 8 Å to 20 Å and 70 ns each for *d*_COM-COM_ = 21 Å to 24 Å. This resulted in a total of ~3.5 μs of MD simulation time.

Within each umbrella sampling window, a harmonic potential with a force constant of 4 kcal mol^−1^ Å^−2^ was applied to restrain the conformations close to the reference point. Force constants of 20 kcal mol^−1^ Å^−2^ were also used to restrain conformations whose initial *d*_COM-COM_ ranged from 8.0 Å to 10 Å to generate approximately Gaussian-shaped frequency distributions. Otherwise, the parameters described above were used for thermalization and production runs. Finally, to compute the errors at the reference points of the PMF profiles, the Monte Carlo bootstrapping analysis implemented in WHAM using 200 resampling trials was applied.

### Estimation of association free energy

An association free energy was estimated from the obtained PMF following the membrane two-body derivation from Johnston *et al*.^54^. In brief, the PMF is integrated along the reaction coordinate to calculate an association constant (*K*_a_), transformed to the mole fraction scale (*K*_x_) taking into account the number of lipids *N*_L_ per surface area *A*, and this value is used to calculate the difference in free energy between dimer and monomers (Δ*G*), according to eqs 1–3

1 $${K}_{a}=\frac{||\Omega ||}{{(2\pi )}^{2}}{\int }_{0}^{D}r{e}^{\frac{-w(r)}{{k}_{B}T}}dr$$

2 $${K}_{x}={K}_{a}\frac{{N}_{L}}{A}$$

3 $${\rm{\Delta }}G=-RT\,ln({K}_{x})$$

where *r* is the value of the reaction coordinate, *w*(*r*) is the PMF at value *r*, *D* is the maximum distance at which the protein is still considered a dimer, *k*_B_ is the Boltzmann constant, and *T* is the temperature at which the simulations were performed. Additionally, a factor that considers the restriction of the configurational space of the monomers upon dimer formation is included in terms of the sampled angle between the two chains in the dimeric state (eq. 4)

4 $$||\Omega ||=[\max ({\theta }_{a})-\,\min ({\theta }_{a})]\,\ast \,[\max ({\theta }_{b})-\,\min ({\theta }_{b})]$$

and the accessible space for the monomers, (2π)^2^. In eq. 4, the angle *θ*_a_ is defined as the angle formed between the vectors connecting the COM of helix 1 with the COM of helix 2 and with the COM of residues V971 to V973 of the latter helix; *θ*_b_ is defined analogously starting from the COM of helix 2 and using the COM of residues L698 to V700 in helix 1.

### Analysis of trajectories

For the analysis of the trajectories, *ptraj*/*cpptraj*^110^ of the AmberTools 14 suite of programs was applied. For the unbiased MD simulations, the first 200 ns were not considered for analysis. To evaluate the helix-helix interface (indicated as *d*_COM-COM-_), a maximal distance of 3.5 Å and a minimal angle of 120° were used as exclusion criteria to identify hydrogen bond formation, as was a maximal distance of 4 Å to identify salt bridge formation. To examine the OMC interface, the distance between the C_α_ atoms of G972/G976 (α_IIb_ subunit) and V700/I704 (β_3_ subunit) was computed (indicated as *d*_OMC_). To evaluate the IMC interface, the distance between the centers of mass of the phenyl rings of F992 or F993 (α_IIb_ subunit) and W715 (β_3_ subunit) (indicated as *d*_IMC1_ and *d*_IMC2_, respectively) was computed, and the smaller distance of the two was considered further as *d*_IMC_. To evaluate the TMD association, we calculated the total number of native and non-native contacts formed between the two helices. A native contact was defined as a contact satisfying the distance cutoff of 7 Å in the first frame (used as reference frame), a non-native contact as a contact satisfying the distance cutoff of 7 Å without being already found in the reference frame. This definition is according to ref.^47^. The same set of analyses was also carried out to analyze the configurations sampled during umbrella sampling. For all the configurations *t* generated, we estimated a weight *w*^*t*^ according to eqs 7 and 8 from ref.^56^. The reweighting is performed over the entire ensemble of each system and, then, normalized by dividing each *w*^*t*^ by the sum of all *w*^*t*^ for each integrin system.

### Statistical analysis

Results from three independent MD simulations are expressed as arithmetic means ± SEM calculated over the time. The overall SEM for each simulated system was calculated according to the law of error propagation (eq. 5):

5 $${{\rm{SEM}}}_{total}=\sqrt{{{\rm{SEM}}}_{1}^{2}+{{\rm{SEM}}}_{2}^{2}+{{\rm{SEM}}}_{3}^{2}}$$

where the subscripts *i* = {1, 2, 3} indicate the three trajectories. SEM_*i*_ was computed following ref.^111^ (see particularly eqs 11–13 there) by, first, detecting the decorrelation time of an investigated variable along each MD simulation and, second, establishing the effective sample size from that time, which is then used to compute SEM_*i*_. In the case of hydrogen bond, salt bridge, and contact analyses, SEM is calculated from the standard deviation (SD) of the three means of the three MD simulations according to eq. 6, assuming that the three MD simulations are statistically independent:

6 $$SEM=SD\,/\sqrt{3}$$

*p* values related to eq. 5 are calculated according to the Student’s *t*-test for parametric testing, with $${N}_{eff}\equiv (T-$$$${t}_{o}+1)/g$$, where $${N}_{eff}$$ is the total number of uncorrelated frames within the trajectories based on the statistical inefficiency *g*_*t0*_ and the simulation time range [*t*_0_, *T*] following ref.^111^; the same test is applied for *p* values related to eq. 6, with *N*_*eff*_ = 3. Differences between mean values are considered to be statistically significant if *p* < 0.05 and *p* < 0.001 (indicated as “*” and “**” in figures, respectively) and highly statistically significant if *p* < 0.0001 (indicated as “***” in figures). The statistical analysis was performed using the R software^112^ and the pymbar module for MBAR^113^.

### Data availability

All data generated or analyzed during this study are included in this published article (and its Supplementary Information files).

## Electronic supplementary material

Supplementary information
