# Supplementary material for: On the contributing role of the transmembrane domain for subunit-specific sensitivity of integrin activation
Source: Sci Rep. 2018 Apr 10;8:5733. doi: 10.1038/s41598-018-23778-5 (PMC5893634; doi:10.1038/s41598-018-23778-5)
Supplement: Supplementary file 1 — Supplementary information [file 41598_2018_23778_MOESM1_ESM.pdf]

Supporting Information

On the contributing role of the transmembrane domain for subunit-specific sensitivity of integrin activation

Giulia Pagani[1], Holger Gohlke[1,2*]

[1]Institute for Pharmaceutical and Medicinal Chemistry, Heinrich-Heine-Universität Düsseldorf, 40225 Düsseldorf, Germany

[2]John von Neumann Institute for Computing (NIC), Jülich Supercomputing Centre (JSC) & Institute for Complex Systems - Structural Biochemistry (ICS 6), Forschungszentrum Jülich GmbH, 52425 Jülich, Germany.

Table of Contents

Supplemental Tables 3

Table S1: OMC and IMC total number of contacts. 3

Table S2: RMSF of the $\alpha_{IIb}\beta_3$, $\alpha v\beta_3$, and $\alpha_5\beta_1$ integrin TMDs. 4

Table S3: OMC and IMC distances. 6

Table S4: Hydrogen bond between $K716_{N\varepsilon}$ and $F992_O$ 6

Table S5: Salt bridge between $K716_{N\varepsilon}$ and the oxygens of phospholipid head groups. . 6

Table S6: Thermodynamic quantities for each TMD system.[a] 7

Table S7: OMC and IMC distances across the umbrella sampling windows linked to free energy minima I – III. 8

Table S8: $d_{COM\text{-}COM}$ distance computed from unbiased MD simulations of $\alpha_{IIb}\beta_3$, $\alpha_v\beta_3$, and $\alpha_5\beta_1$ integrin. 9

Supplemental Figures 10

Fig S1: Assessment of the quality of TMD homology models. 10

Fig S2: Convergence of internal motions in unbiased MD simulations. 11

Fig S3: Total number of contacts calculated for $\alpha_{IIb}\beta_3$, $\alpha_v\beta_3$, and $\alpha_5\beta_1$ integrin. 12

Fig S4: Overlap of umbrella sampling simulations of the association of TMDs of (A) $\alpha_{IIb}\beta_3$, (B) $\alpha_v\beta_3$, and (C) $\alpha_5\beta_1$ integrin. 13

Fig S5: Convergence of the PMFs of the association of TMDs of (A, A') $\alpha_{IIb}\beta_3$, (B, B') $\alpha_v\beta_3$, and (C, C') $\alpha_5\beta_1$ integrin. 14

Fig S6: Reweighting of configurations obtained by umbrella sampling of the TMDs of (A) $\alpha_{IIb}\beta_3$, (B) $\alpha_v\beta_3$, and (C) $\alpha_5\beta_1$ integrin. 15

Fig S7: $d_{COM\text{-}COM}$ distance computed from unbiased MD simulations of $\alpha_{IIb}\beta_3$, $\alpha_v\beta_3$, and $\alpha_5\beta_1$ integrin. 15

Supplemental References 16

Supplemental Tables

Table S1: OMC and IMC total number of contacts.

	**Residue number α subunit**	**SimI[a]**	**SimII[a]**	**SimIII[a]**	**Mean ± SEM[b]**
**$\alpha_{IIb}\beta_3$ OMC**	972	408	452	295	385 ± 5.19
	973	424	513	456	464 ± 3.88
	974	190	80	49	106 ± 4.97
	975	147	184	135	155 ± 2.92
	976	390	426	366	394 ± 3.17
	**Total**	1559[c]	1655[c]	1301[c]	**1505 ± 7.81**[d]
**$\alpha_v\beta_3$ OMC**	972	611	375	356	447 ± 6.88
	973	520	380	161	353 ± 7.77
	974	49	24	8	27 ± 2.62
	975	231	47	63	113 ± 5.83
	976	375	151	170	232 ± 6.43
	**Total**	1786[c]	977[c]	758[c]	**1174 ± 13.43**[d]
**$\alpha_5\beta_1$ OMC**	972	266	367	289	307 ± 4.20
	973	256	197	52	168 ± 5.91
	974	13	12	2	9 ± 1.42
	975	69	264	217	183 ± 5.82
	976	178	224	138	180 ± 3.79
	**Total**	782[c]	1064[c]	698[c]	**848 ± 7.99**[d]
**$\alpha_{IIb}\beta_3$ IMC**	992	731	583	45	453 ± 10.97
	993	932	1021	1011	988 ± 3.88
	**Total**	1663[c]	1604[c]	1056[c]	**1441 ± 10.56**[d]
**$\alpha_v\beta_3$ IMC**	992	586	662	751	666 ± 5.25
	993	1014	860	169	681 ± 12.25
	**Total**	1600[c]	1522[c]	920[c]	**1347 ± 11.14**[d]
**$\alpha_5\beta_1$ IMC**	992	640	641	37	439 ± 10.78
	993	840	972	962	925 ± 4.95
	**Total**	1480[c]	1613[c]	999[c]	**1364 ± 10.38**[d]

[a] Mean values calculated for each simulation per residue.
[b] Mean value and SEM (eq. 6 in the main text) calculated over three simulations per residue.
[c] Total number of contacts at the OMC/IMC calculated for each simulation.
[d] Mean value and SEM calculated over three simulations.

Table S2: RMSF of the $\alpha_{IIb}\beta_3$, $\alpha v\beta_3$, and $\alpha_5\beta_1$ integrin TMDs.

Residue number α-subunit	$\alpha_{IIb}\beta_3$[a]	$\alpha_v\beta_3$[a]	$\alpha_5\beta_1$[a]	Min value[b]
959	4.52	6.29	4.73	4.52
960	4.18	5.01	3.76	3.76
961	3.35	4.21	3.18	3.18
962	2.59	3.32	2.89	2.59
963	1.93	2.77	2.95	1.93
964	1.45	2.08	2.43	1.45
965	1.17	1.56	1.77	1.17
966	0.99	1.17	1.62	0.99
967	0.92	1.12	1.47	0.92
968	0.73	1.03	1.20	0.73
969	0.65	0.78	1.07	0.65
970	0.70	0.73	0.99	0.70
971	0.65	0.70	0.89	0.65
972	0.54	0.62	0.82	0.54
973	0.61	0.62	0.77	0.61
974	0.67	0.65	0.72	0.65
975	0.62	0.63	0.68	0.62
976	0.57	0.62	0.64	0.57
977	0.61	0.64	0.64	0.61
978	0.62	0.66	0.66	0.62
979	0.57	0.65	0.64	0.57
980	0.55	0.63	0.61	0.55
981	0.57	0.65	0.63	0.57
982	0.58	0.70	0.67	0.58
983	0.58	0.72	0.64	0.58
984	0.56	0.67	0.60	0.56
985	0.58	0.72	0.67	0.58
986	0.68	0.83	0.75	0.68
987	0.73	0.86	0.71	0.71
988	0.77	0.96	0.77	0.77
989	0.94	1.16	0.96	0.94
990	1.09	1.28	1.04	1.04
991	1.11	1.32	1.06	1.06
992	1.12	1.28	1.00	1.00
993	1.30	1.76	1.21	1.21
994	1.63	2.20	1.61	1.61
995	2.51	2.51	2.33	2.33
996	3.45	3.22	3.50	3.22
997	4.42	4.14	5.16	4.14
998	5.70	5.80	7.08	5.70
999	6.55	6.81	8.40	6.55

[a] Mean values, in Å, of the backbone atoms after a mass-weighted alignment onto the TM segment embedded in the membrane (residues P996-V1015 and D718-I747, for the α_{IIb}- and β_3-subunits, respectively) calculated over three MD simulations for $\alpha_{IIb}\beta_3$, $\alpha_v\beta_3$, and $\alpha_5\beta_1$ integrin.
[b] Minimal value for each row. Red color highlights when the minimal value corresponds to the value for $\alpha_{IIb}\beta_3$ integrin.

Table S2 continued.

Residue number β-subunit	$\alpha_{IIb}\beta_3$[a]	$\alpha_v\beta_3$[a]	$\alpha_5\beta_1$[a]	Min value[b]
684	13.17	13.77	11.09	11.09
685	12.41	13.01	10.26	10.26
686	10.30	11.23	8.76	8.76
687	8.31	9.64	7.34	7.34
688	6.31	8.19	5.59	5.59
689	4.70	6.18	4.28	4.28
690	3.20	5.13	3.23	3.20
691	2.00	3.66	2.51	2.00
692	1.50	2.15	2.09	1.50
693	0.93	1.42	1.58	0.93
694	0.85	1.08	1.30	0.85
695	0.87	0.99	1.26	0.87
696	0.72	0.88	1.15	0.72
697	0.55	0.70	0.87	0.55
698	0.61	0.66	0.83	0.61
699	0.62	0.71	0.83	0.62
700	0.53	0.64	0.79	0.53
701	0.53	0.62	0.76	0.53
703	0.67	0.78	0.80	0.67
704	0.63	0.76	0.70	0.63
705	0.62	0.71	0.70	0.62
706	0.67	0.70	0.75	0.67
707	0.68	0.68	0.78	0.68
708	0.63	0.68	0.74	0.63
709	0.61	0.70	0.70	0.61
710	0.61	0.64	0.70	0.61
711	0.64	0.62	0.74	0.62
712	0.63	0.79	0.79	0.63
713	0.69	0.87	0.85	0.69
714	0.81	0.88	0.89	0.81
715	0.85	1.01	0.91	0.85
716	1.02	1.21	1.09	1.02
717	1.39	1.35	1.27	1.27
718	1.63	1.59	1.43	1.43
719	1.87	1.90	1.72	1.72
720	2.43	2.20	2.25	2.20
721	3.12	2.45	2.61	2.45
722	3.19	2.72	2.70	2.70
723	3.26	3.34	2.94	2.94
724	4.03	3.76	3.55	3.55
725	4.75	4.46	3.64	3.64
726	5.49	5.35	3.67	3.67
727	6.57	6.73	4.31	4.31

[a] Mean values, in Å, of the backbone atoms after a mass-weighted alignment onto the TM segment embedded in the membrane (residues P996-V1015 and D718-I747, for the α_{IIb}- and β_3-subunits, respectively) calculated over three MD simulations for $\alpha_{IIb}\beta_3$, $\alpha_v\beta_3$, and $\alpha_5\beta_1$ integrin.
[b] Minimal value for each row. Red color highlights when the minimal value corresponds to the value for $\alpha_{IIb}\beta_3$ integrin.

Table S3: OMC and IMC distances.

	SimI[a]	SimII[a]	SimIII[a]	Mean± SEM[b]
$d_{OMC}\alpha_{IIb}\beta_3$	6.9 ± 0.01	6.9 ± 0.01	6.8 ± 0.02	6.8 ± 0.03
$d_{OMC}\alpha_v\beta_3$	7.2 ± 0.03	7.2 ± 0.05	7.1 ± 0.02	7.1 ± 0.06
$d_{OMC}\alpha_5\beta_1$	10.1 ± 0.3	7.6 ± 0.06	7.5 ± 0.02	8.4 ± 0.09
$d_{IMC}\alpha_{IIb}\beta_3$	7.5 ± 0.1	5.0 ± 0.2	7.1 ± 0.02	6.8 ± 0.5
$d_{IMC}\alpha_v\beta_3$	6.2 ± 0.3	6.7 ± 0.9	6.0 ± 0.1	6.3 ± 0.9
$d_{IMC}\alpha_5\beta_1$	6.0 ± 0.1	6.1 ± 0.1	5.7 ± 0.05	5.9 ± 0.2

[a] Mean values and SEM, in Å, calculated within each simulation.
[b] Mean value and SEM (eq. 5 in the main text), in Å, calculated over three simulations.

Table S4: Hydrogen bond between $K716_{N\varepsilon}$ and $F992_O$.

	SimI[a]	SimII[a]	SimIII[a]	Mean ± SEM[b]
$\alpha_{IIb}\beta_3$ TMD	74	40	0	38 ± 21.4
$\alpha_v\beta_3$ TMD	71	26	13	37 ± 17.6
$\alpha_5\beta_1$ TMD	4	61	11	25 ± 17.9

[a] Mean values, in %, calculated for each simulation.
[b] Mean value and SEM (eq. 6 in the main text), in %, calculated over three simulations.

Table S5: Salt bridge between $K716_{N\varepsilon}$ and the oxygens of phospholipid head groups.

	SimI[a]	SimII[a]	SimIII[a]	Mean ± SEM[b]
$\alpha_{IIb}\beta_3$ TMD	55	59	82	65 ± 8.4
$\alpha_v\beta_3$ TMD	21	45	49	38 ± 8.7
$\alpha_5\beta_1$ TMD	9	9	60	26 ± 17.0

[a] Mean values, in %, calculated for each simulation.
[b] Mean value and SEM (eq. 6 in the main text), in %, calculated over three simulations.

Table S6: Thermodynamic quantities for each TMD system.[a]

System	$\alpha_{IIb}\beta_3$ TMD	$\alpha_v\beta_3$ TMD	$\alpha_5\beta_1$ TMD
$\|\|\Omega\|\|$[a]	0.07	0.06	0.1
K_a[b]	140733.1	1360.9	75.2
K_X	2010.5	19.4	1.1
ΔG[c]	-4.5	-1.8	-0.04

[a] The integral in eq. 1 in the main text was evaluated at D = 14 Å.
[b] In radians.
[c] In $Å^2$.
[d] In kcal mol^{-1}.

Table S7: OMC and IMC distances across the umbrella sampling windows linked to free energy minima I – III.

Distance	Minima I			Mean ± SEM[b]
	w3[a]	w4[a]	w5[a]	
$d_{OMC}\alpha_{IIb}\beta_3$	6.9 ± 0.01	7.5 ± 0.05	7.4 ± 0.07	7.3 ± 0.09
$d_{OMC}\alpha_v\beta_3$	7.2 ± 0.05	7.5 ± 0.03	7.6 ± 0.07	7.4 ± 0.09
$d_{OMC}\alpha_5\beta_1$	7.3 ± 0.04	9.2 ± 0.05	7.6 ± 0.02	8.1 ± 0.09
$d_{IMC}\alpha_{IIb}\beta_3$	4.2 ± 0.07	4.8 ± 0.03	6.2 ± 0.02	5.1 ± 0.08
$d_{IMC}\alpha_v\beta_3$	4.3 ± 0.03	8.0 ± 0.3	5.6 ± 0.1	6.0 ± 0.3
$d_{IMC}\alpha_5\beta_1$	6.0 ± 0.5	6.8 ± 0.3	5.1 ± 0.01	6.0 ± 0.6

Distance	Minima II		Mean ± SEM[b]
	w8[a]	w9[a]	
$d_{OMC}\alpha_{IIb}\beta_3$	10.9 ± 0.1	9.3 ± 0.08	10.1 ± 0.03
$d_{OMC}\alpha_v\beta_3$	10.8 ± 0.07	9.9 ± 0.04	10.4 ± 0.09
$d_{OMC}\alpha_5\beta_1$	11.4 ± 0.1	10.4 ± 0.02	10.9 ± 0.1
$d_{IMC}\alpha_{IIb}\beta_3$	6.1 ± 0.05	5.0 ± 0.07	5.6 ± 0.08
$d_{IMC}\alpha_v\beta_3$	6.2 ± 0.1	6.2 ± 0.4	6.2 ± 0.4
$d_{IMC}\alpha_5\beta_1$	6.2 ± 0.2	9.7 ± 0.4	8.0 ± 0.4

Distance	Minima III		Mean ± SEM[b]
	w11[a]	w12[a]	
$d_{OMC}\alpha_{IIb}\beta_3$	15.7 ± 0.05	13.1 ± 0.06	14.4 ± 0.08
$d_{OMC}\alpha_v\beta_3$	13.7 ± 0.1	13.1 ± 0.1	13.4 ± 0.1
$d_{OMC}\alpha_5\beta_1$	13.4 ± 0.1	16.7 ± 0.2	15.0 ± 0.03
$d_{IMC}\alpha_{IIb}\beta_3$	5.8 ± 0.3	7.9 ± 0.3	6.8 ± 0.4
$d_{IMC}\alpha_v\beta_3$	8.0 ± 0.3	7.1 ± 0.08	7.5 ± 0.3
$d_{IMC}\alpha_5\beta_1$	6.1 ± 0.1	5.5 ± 0.05	5.8 ± 0.1

[a] Mean value and SEM, in Å, calculated within the respective umbrella sampling window.
[b] Mean value and SEM (eq. 5 in the main text), in Å, calculated over two resp. three umbrella sampling windows.

Table S8: $d_{COM\text{-}COM}$ distance computed from unbiased MD simulations of $\alpha_{IIb}\beta_3$, $\alpha_v\beta_3$, and $\alpha_5\beta_1$ integrin.

	SimI[a]	SimII[a]	SimIII[a]	Mean ± SEM[b]
**$\alpha_{IIb}\beta_3$ TMD**	9.2 ± 0.06	8.9 ± 0.07	10 ± 0.05	9.3 ± 0.1
**$\alpha_v\beta_3$ TMD**	9.5 ± 0.16	9.8 ± 0.23	9.5 ± 0.07	9.6 ± 0.3
**$\alpha_5\beta_1$ TMD**	9.4 ± 0.07	10.8 ± 0.27	10.4 ± 0.16	10.2 ± 0.3

[a] Mean values and SEM, in Å, calculated for each simulation.
[b] Mean value and SEM (eq. 5 in the main text), in Å, calculated over three simulations.

Supplemental Figures

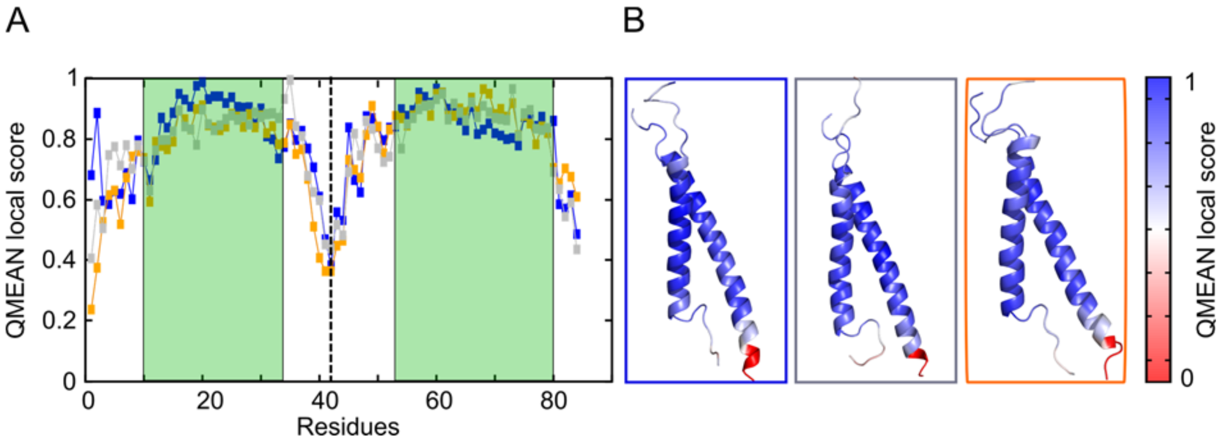

Fig S1: Assessment of the quality of TMD homology models.

The QMEANBrane version of the QMEAN scoring function implemented within the QMEAN server is used to evaluate the quality of homology models of TMDs of $\alpha_v\beta_3$ and $\alpha_5\beta_1$ integrin, respectively, in comparison to the NMR structure of $\alpha_{IIb}\beta_3$ (PDB ID: 2K9J) used as a template[1,2]. The local scores are provided in a range [0,1], with one being the optimal. (**A**) Local scores computed for the three structures (blue: $\alpha_{IIb}\beta_3$ TMD; gray: $\alpha_v\beta_3$ TMD; orange: $\alpha_5\beta_1$ TMD). Two green boxes are used to highlight the TM region (residues P996-V1015 and D718-I747 for the α_{IIb}- and β_3-subunits, respectively), and the black dashed line separates the α-subunit (left side) from the β-subunit (right side). (**B**) In each box (colored as in panel A), the QMEAN local scores are encoded in a color gradient from red to blue and mapped onto the integrin TMDs depicted in cartoon representation.

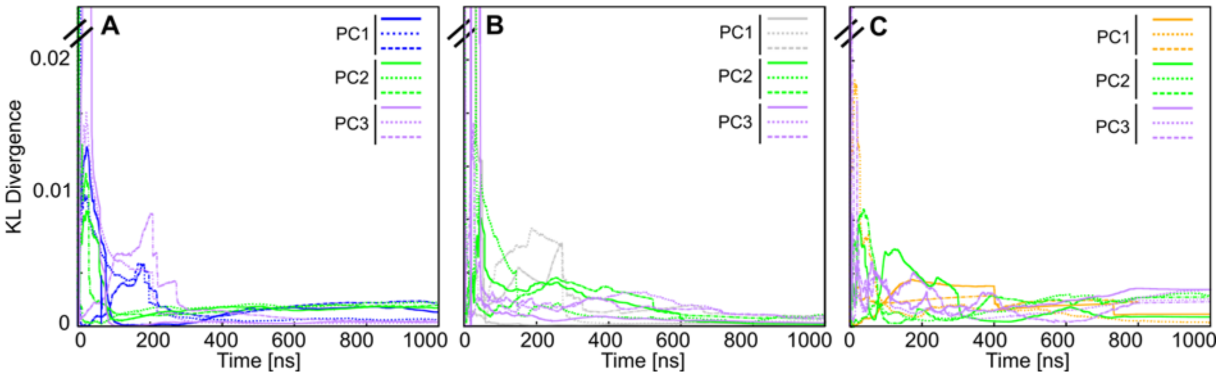

Fig S2: Convergence of internal motions in unbiased MD simulations.

Kullback-Leibler (KL) divergence[3] as a parameter to evaluate the convergence of the three independent MD simulations of (**A**) $\alpha_{IIb}\beta_3$ integrin TMD, (**B**) $\alpha_v\beta_3$ integrin TMD, and (**C**) $\alpha_5\beta_1$ integrin TMD. The KL divergence was computed for pair-wise comparisons of replicates of MD simulations in terms of histograms describing the projection of snapshots onto a given principal component (PC). The principal component analysis was performed on the residues embedded in the membrane (as shown in Fig. S1) after alignment onto their C_α atoms; color code of PC1 as in Fig. S1, PC2 colored in green and PC3 in purple in all plots.

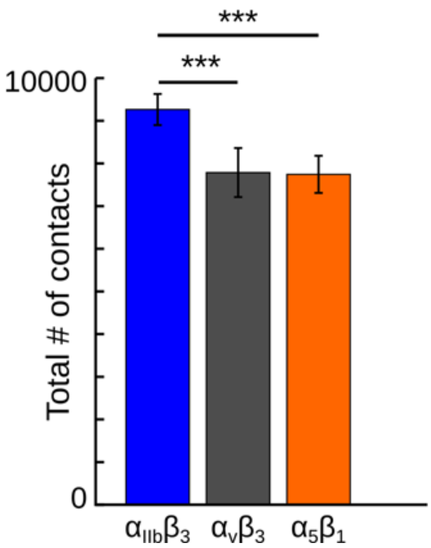

Fig S3: Total number of contacts calculated for $\alpha_{IIb}\beta_3$, $\alpha_v\beta_3$, and $\alpha_5\beta_1$ integrin.

Histograms of the total number of contacts ("native" and "non-native") between the α- and β-subunits averaged over three independent MD simulations for TMDs of $\alpha_{IIb}\beta_3$ (blue), $\alpha_v\beta_3$ (grey), and $\alpha_5\beta_1$ (orange) integrin, respectively. Error bars denote the SEM (eq. 6 in the main text). Stars denote a significant difference ($p < 0.0001$) between the TMDs of $\alpha_{IIb}\beta_3$ and $\alpha_v\beta_3$, and the TMDs of $\alpha_{IIb}\beta_3$ and $\alpha_5\beta_1$, respectively. A native contact was defined as a contact between two residues satisfying a distance cut-off of 7 Å in the first (reference) frame of a MD trajectory; a non-native contact is any contact whose distance satisfies the distance cut-off of 7 Å without being already found in the reference frame. This definition is according to ref.[4].

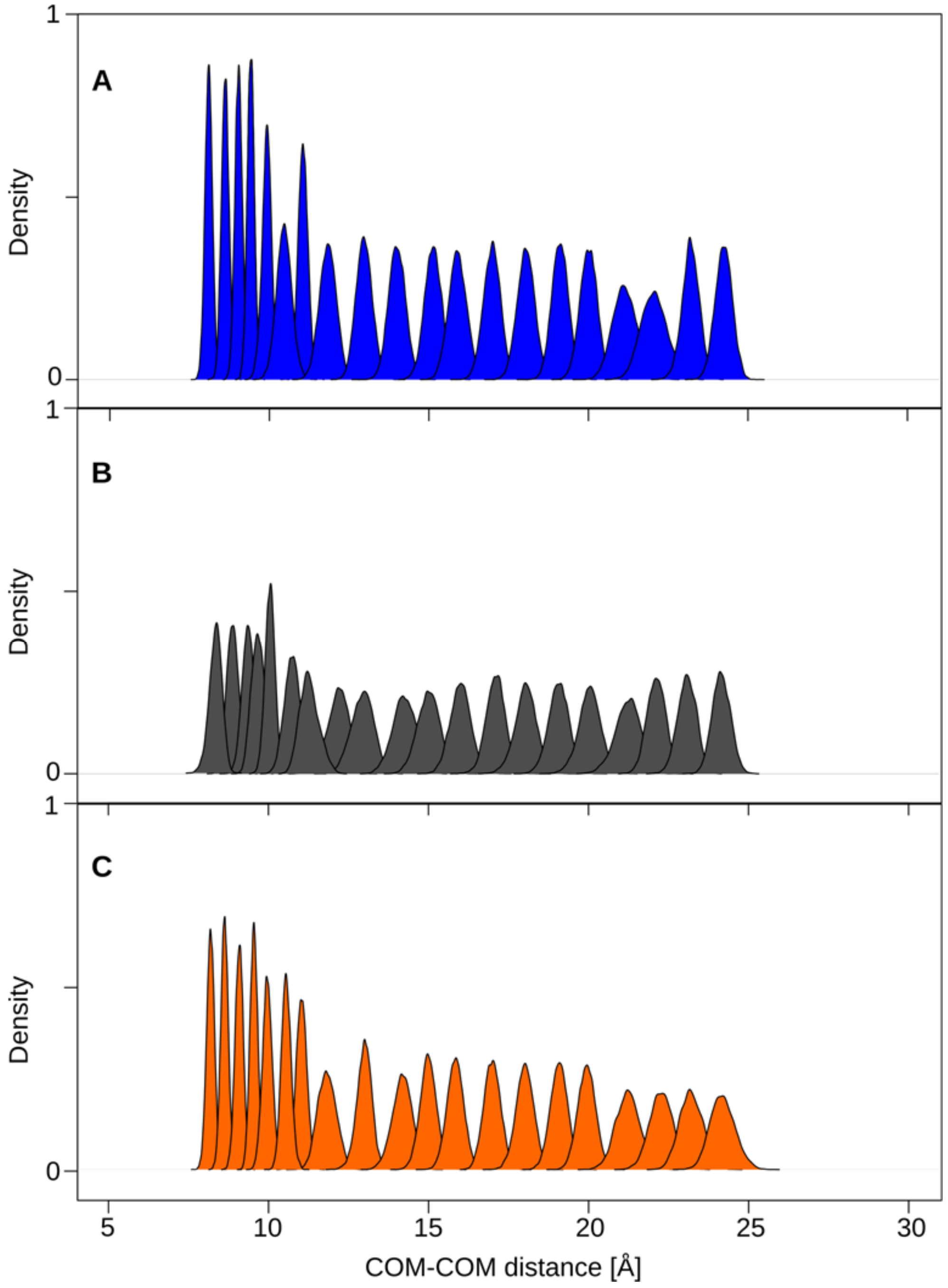

Fig S4: Overlap of umbrella sampling simulations of the association of TMDs of (A) $\alpha_{IIb}\beta_3$, (B) $\alpha_v\beta_3$, and (C) $\alpha_5\beta_1$ integrin.

Frequency distributions of values of the reaction coordinate obtained from umbrella sampling simulations.

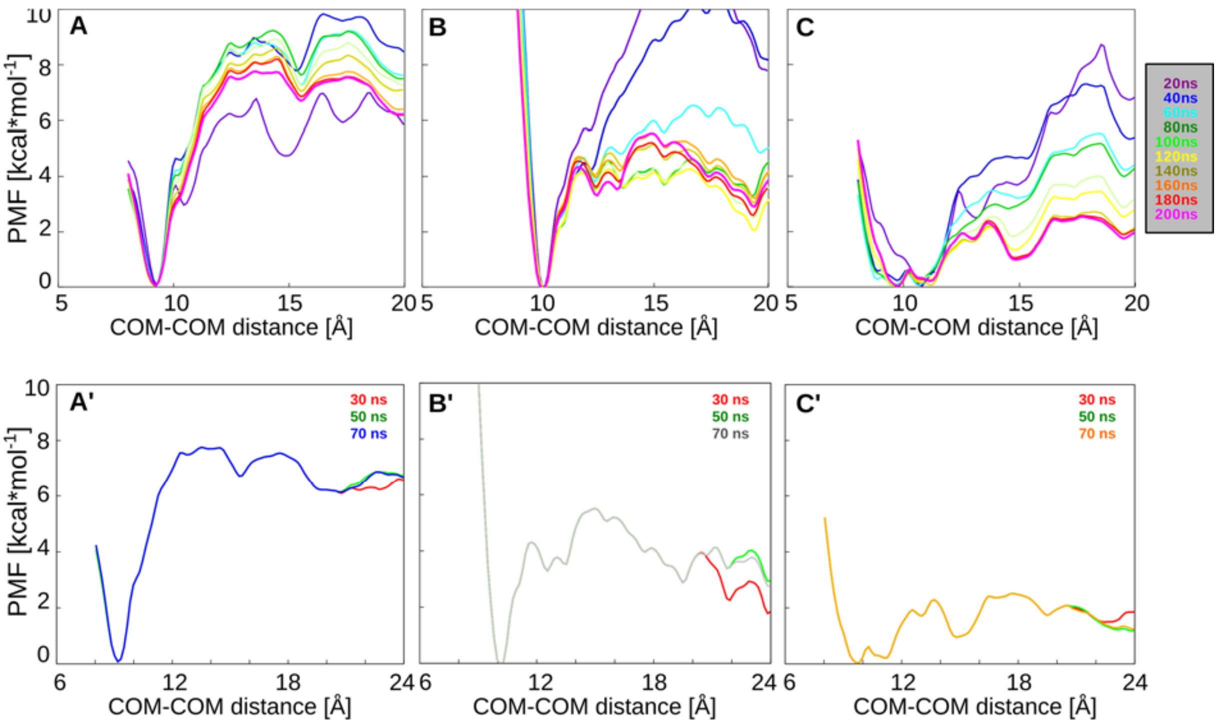

Fig S5: Convergence of the PMFs of the association of TMDs of (A, A') $\alpha_{IIb}\beta_3$, (B, B') $\alpha_v\beta_3$, and (C, C') $\alpha_5\beta_1$ integrin.

(**A-C**) Development over time of the PMF profiles computed for the association of the integrin's TMDs as indicated by the color scale at the right. In all cases, the PMF curves almost perfectly overlap after 160 ns of sampling time per umbrella sampling window. The reaction coordinate (distance between the centers of mass of the α- and β-subunits) ranges from 8 Å (fully associated state) to 20 Å (dissociated state). (**A'-C'**) Development over time of the PMF profiles computed for the full disassociation of the integrin's TMDs as indicated by the color scale inside each plot. In all cases, an almost perfect overlap is reached after 50 ns of sampling time per umbrella sampling window. The reaction coordinate considered ranges from 21 Å to 24 Å (fully dissociated state).

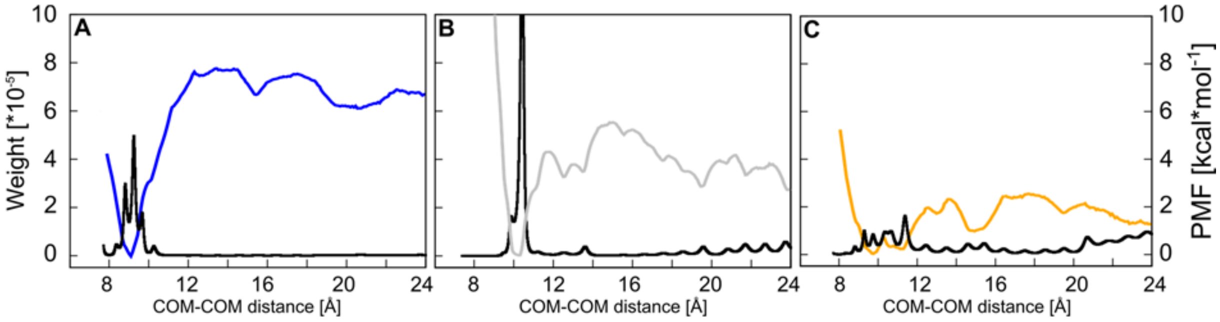

Fig S6: Reweighting of configurations obtained by umbrella sampling of the TMDs of (A) $\alpha_{IIb}\beta_3$, (B) $\alpha_v\beta_3$, and (C) $\alpha_5\beta_1$ integrin.

The curves colored black (left ordinate values) depict the weight w^t of a configuration t computed according to eqs. 7 and 8 from ref.[5], which is normalized by dividing it by the sum of all weights for each integrin system. Overlaid is the respective PMF profile (colored blue, grey, or orange; right ordinate values). The reweighting is performed considering the entire ensemble for each system between reaction coordinate values 8 to 24 Å.

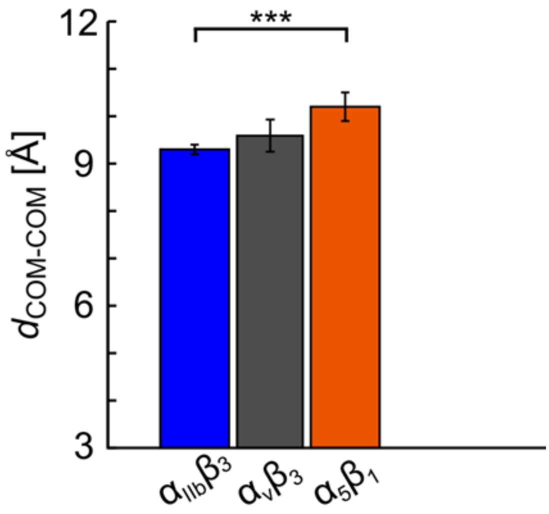

Fig S7: $d_{COM-COM}$ distance computed from unbiased MD simulations of $\alpha_{IIb}\beta_3$, $\alpha_v\beta_3$, and $\alpha_5\beta_1$ integrin.

Histograms of the distance $d_{COM-COM}$ (see main text for definition) averaged over three MD simulations. Values for $\alpha_{IIb}\beta_3$, $\alpha_v\beta_3$, and $\alpha_5\beta_1$ TMD are represented in blue, grey, and orange, respectively. Error bars denote the SEM (eq. 5 in the main text). Stars denote a significant difference ($p < 0.0001$) between the TMDs of $\alpha_{IIb}\beta_3$ and $\alpha_5\beta_1$.

Supplemental References

1 Lau, T. L., Kim, C., Ginsberg, M. H. & Ulmer, T. S. The structure of the integrin alpha(IIb)beta(3) transmembrane complex explains integrin transmembrane signalling. *EMBO J.* **28**, 1351-1361, (2009).

2 Studer, G., Biasini, M. & Schwede, T. Assessing the local structural quality of transmembrane protein models using statistical potentials (QMEANBrane). *Bioinformatics* **30**, I505-I511, (2014).

3 Galindo-Murillo, R., Roe, D. R. & Cheatham, T. E., 3rd. Convergence and reproducibility in molecular dynamics simulations of the DNA duplex d(GCACGAACGAACGAACGC). *Biochim. Biophys. Acta* **1850**, 1041-1058, (2015).

4 Case, D. A., et al. The Amber biomolecular simulation programs. *J. Comp. Chem.* **26**, 1668-1688, (2005).

5 Moradi, M., Enkavi, G. & Tajkhorshid, E. Atomic-level characterization of transport cycle thermodynamics in the glycerol-3-phosphate: phosphate antiporter. *Nat. Commun.* **6**, 8393; 10.1038/ncomms9393, (2015).
